# Supplementary material for: Quantum behavior of the Duffing oscillator at the dissipative phase transition
Source: Nat Commun. 2023 May 20;14:2896. doi: 10.1038/s41467-023-38217-x (PMC10199948; doi:10.1038/s41467-023-38217-x)
Supplement: Supplementary file 1 — Supplementary Information [file 41467_2023_38217_MOESM1_ESM.pdf]

Supplemental Information for
"Quantum behavior of the Duffing oscillator at the dissipative phase transition"

Qi-Ming Chen,[1,2] Michael Fischer,[1,2] Yuki Nojiri,[1,2] Michael Renger,[1,2] Edwar Xie,[1,2] Matti Partanen,[1,*] Stefan Pogorzalek,[1,2,†] Kirill G. Fedorov,[1,2] Achim Marx,[1] Frank Deppe,[1,2,3,†] and Rudolf Gross[1,2,3]

[1] *Walther-Meißner-Institut, Bayerische Akademie der Wissenschaften, 85748 Garching, Germany*
[2] *Physik-Department, Technische Universität München, 85748 Garching, Germany*
[3] *Munich Center for Quantum Science and Technology (MCQST), 80799 Munich, Germany*
(Dated: April 2, 2023)

CONTENTS

Supplementary Note 1. Model and theory 1
A. Description of the system 1
B. Quantum theory of the Duffing oscillator 2
C. Classical theory of the Duffing oscillator 2

Supplementary Note 2. System characterization 3
A. Experimental setup 3
B. Control and readout modules 4
C. Closed-system parameters 4
D. Open-system parameters 6
E. Gain and noise in the output path 7
F. Attenuation and offset in the input path 8

Supplementary Note 3. Experimental methods 9
A. Initial state preparation 9
B. Pulsed heterodyne measurement 10
C. Photon correlations 10

Supplementary Note 4. Supplementary data 11
A. Closure of the hysteresis loop in the long-time limit 11
B. Extracting the Liouvillian gap from time-domain measurements 11
C. Quantum state tomography of the phase transition process 12
D. Squeezing levels in the two phases besides the phase transition 13
E. The dephasing effect and possible two-photon processes 14

* Present address: IQM, Keilaranta 19, FI-02150 Espoo, Finland
† Present address: IQM, Nymphenburger Str. 86, 80636 Munich, Germany

Supplementary Note 1. MODEL AND THEORY

A. Description of the system

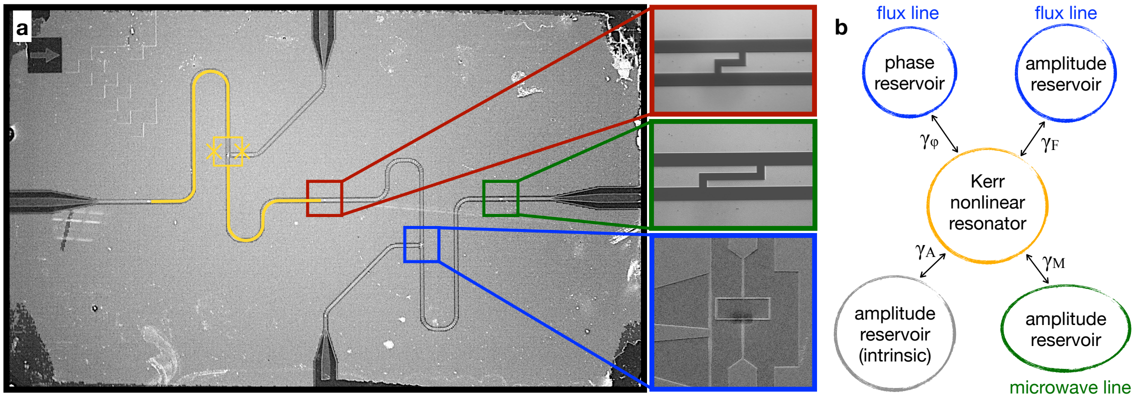

Supplementary Fig. 1: Optical photograph of the sample chip. (a) The sample consists of two transmission line resonators with a DC-SQUID embedded in the middle (yellow). The two resonators are coupled by a finger capacitor, as shown in the top red box. They are also coupled to two external feedlines, as shown in the middle green box. The resonant frequency and the nonlinearity of each individual resonator is controlled by the T-shaped flux control lines, as shown in the bottom blue box. **(b)** Schematic of the entire system, where the resonator is coupled to different baths.

An optical photograph of a reference sample is shown in Supplementary Fig. 1a, which has the same design as that used in the experiment. The sample is fabricated on a 525 μm-thick silicon chip with an area of $10 \times 6\,\mathrm{mm}^2$ using double-angle shadow evaporation and lift-off procedures. The superconductor layer is made of aluminum with a thickness of 140 nm. The major part of the sample consists of two 7.2 mm-long and 13.2 μm-wide transmission line resonators with two DC-SDUIDs embedded in the middle, respectively. The areas of the two SQUIDs are designed to be $10.5 \times 24.5\,\mu\mathrm{m}^2$ and the two junctions in the SQUID loop differ in size to achieve a SQUID asymmetry of approximately 0.13. In addition, two T-shaped on-chip antennae are placed in proximity to the two SQUIDs, respectively, to control the magnetic flux threading the SQUID loops. With this sample design we implement two nonlinear resonators with tunable frequency and nonlinearity [1, 2]. The two resonators are coupled by a 20 μm-long finger capacitor. Furthermore, they are coupled to the outside fields, respectively, by two 40 μm-long finger capacitors at the two ends, and also to the microwave fields in the flux control lines through the two antennae.

In our experiment, we focus on a single resonator which is labelled as “Resonator-2” in the rest of the context. The other resonator is detuned by at least 100 MHz throughout our experiment, which is much larger than the coupling strength between the two resonators (~ 5 MHz) and thus can be fairly neglected. Supplementary Fig. 1b shows the schematic of the whole system, of which the Hamiltonian is described as

$$\begin{aligned} H/\hbar = {} & \omega_A a^\dagger a + U a^\dagger a^\dagger a a + \sum_{k=-\infty}^{+\infty} \omega_k b^\dagger_{A,k} b_{A,k} + i\kappa_A \left(b^\dagger_{A,k} a - b_{A,k} a^\dagger \right) \\ & + \sum_{k=-\infty}^{+\infty} \omega_k b^\dagger_{M,k} b_{M,k} + i\kappa_M \left(b^\dagger_{M,k} a - b_{M,k} a^\dagger \right) \\ & + \sum_{k=-\infty}^{+\infty} \omega_k b^\dagger_{F,k} b_{F,k} + i\kappa_F \left(b^\dagger_{F,k} a - b_{F,k} a^\dagger \right) + i\kappa_\varphi \left(b^\dagger_{F,k} - b_{F,k} \right) a^\dagger a. \end{aligned} \tag{1}$$

Here, a, and $b_{A/M/F,k}$ are the field operators of the resonator and the intrinsic, microwave-line, and flux-line reservoirs, respectively. The parameter $\kappa_{A/M/F/\phi}$ describes the coupling strength between the system, i.e., the resonator, and the corresponding reservoirs. Following the standard derivation of the input-output relation [3, 4] and restricting our discussion to a narrow bandwidth around the driving frequency, ω_d, we obtain the following Heisenberg-Langevin equation for the resonator degree of freedom

$$\dot{a}(t) = -i\omega_A a(t) - i2U a^\dagger(t) a^2(t) - \frac{\gamma_A + \gamma_M + \gamma_F}{2} a(t) - \sqrt{\gamma_M} b^{(M)}_{in}(t) - \sqrt{\gamma_F} b^{(F)}_{in}(t) - \sqrt{\gamma_\varphi} a(t) \left(b^{(F)}_{in}(t) - b^{(F)\dagger}_{in}(t) \right), \tag{2}$$

where $\sqrt{\gamma_{\mathrm{A/M/F}/\varphi}} = \sqrt{2\pi}\kappa_{\mathrm{A/M/F}/\varphi}$. Here, we have assumed the intrinsic amplitude reservoir to be in the vacuum state, and omitted the two-photon loss term by rotating wave approximation (RWA). The input fields are defined as $b_{\mathrm{in}}^{(\mathrm{M/F})}(t) = \int_{-\infty}^{+\infty} d\omega e^{-i\omega t} b_{\omega}^{(\mathrm{M/F})}(0)/\sqrt{2\pi}$, while the output field in the microwave line is $b_{\mathrm{out}}^{(\mathrm{M})}(t) = b_{\mathrm{in}}^{(\mathrm{M})}(t) + \sqrt{\gamma_{\mathrm{M}}}a(t)$.

B. Quantum theory of the Duffing oscillator

Because our experiments are performed in the regime where the dephasing rate, γ_ϕ, is smaller than the total energy dissipation rate, $\gamma = \gamma_{\mathrm{A}} + \gamma_{\mathrm{M}} + \gamma_{\mathrm{F}}$, we temporarily omit the dephasing effect in the following discussions. The experimentally determined values of the sample parameters are reported in Supplementary Note 2 D. We will discuss the dephasing effect and also the possible two-photon processes in Supplementary Note 4 E for achieving a better understanding between the experimental data and the simulation results. In the rotating frame at the driving frequency, ω_{d}, we obtain the simplified Heisenberg-Langevin equation as

$$\dot{a}(t) = -i\Delta a(t) - i2Ua^\dagger(t)a^2(t) - \frac{\gamma}{2}a(t) - \xi. \tag{3}$$

Here, $\Delta = \omega_{\mathrm{A}} - \omega_{\mathrm{d}}$ is the frequency detuning between the resonator and the drive. Besides, we have assumed the input field to be coherent such that we can use a complex number to describe the driving strength, $\xi = -i\sqrt{\gamma_{\mathrm{M/F}}}\langle b_{\mathrm{in}}^{(\mathrm{M/F})}\rangle$ [5]. In most measurements reported in the main text, we drive the sample through the flux line while measuring through the microwave line, in order to avoid the reflecting driving field in the output path.

The above equation describes a quantum-mechanical Duffing oscillator. It has been proven that all orders of signal moments for the steady state (SS) can be calculated in an analytical way [6]

$$\langle a^{\dagger j} a^k \rangle = d^{*j} d^k \frac{\Gamma(c)\Gamma(c^*)_0\mathcal{F}_2\left(k + c, j + c^*, 2\left|d\right|^2\right)}{\Gamma(k+c)\Gamma(j+c^*)_0\mathcal{F}_2\left(c, c^*, 2\left|d\right|^2\right)}. \tag{4}$$

Here, we have used the abbreviations $c = (\Delta - i\gamma/2)/U$ and $d = -\xi/U$. Moreover, ${}_0\mathcal{F}_2(x, y, z) = \sum_{n=0}^{\infty} \Gamma(x)\Gamma(y)z^n/[\Gamma(x+n)\Gamma(y+n)n!]$ is the generalized hypergeometric function, where $\Gamma(\cdot)$ is the gamma special function. This formula indicates that the signal moments of the SS are single valued in the entire parameter space, such that a quantum-mechanical Duffing oscillator does not exhibit bistability or hysteresis [6]. Theoretical calculations also indicate the following Wigner quasi-distribution function of the unique SS [7, 8]

$$W(\alpha, \alpha^*) = \mathcal{N} e^{-2|\alpha|^2} \left|{}_0\mathcal{F}_1(c, 2d\alpha^*)\right|^2, \tag{5}$$

where $\mathcal{N}$ is a normalization factor and ${}_0\mathcal{F}_1(x, z) = \sum_{n=0}^{\infty} \Gamma(x)z^n/[\Gamma(x+n)n!]$ is the hypergeometric function.

C. Classical theory of the Duffing oscillator

If we take the mean value of the Heisenberg-Langevin equation, i.e., Supplementary Eq. (3), and neglect the photon correlations in the third-order term, i.e., $\langle a^\dagger(t)a^2(t)\rangle \to \alpha^*(t)\alpha^2(t)$ where $\alpha(t) = \langle a\rangle$, we obtain the classical equation of motion for the Duffing oscillator [9]

$$\dot{\alpha}(t) = -i\Delta\alpha(t) - i2U\alpha^*(t)\alpha^2(t) - \frac{\gamma}{2}\alpha(t) - \xi(t). \tag{6}$$

One can prove that this equation is equivalent to the celebrated Duffing equation under RWA [10–12]. The SS solution of the classical system can be obtained by solving the following equation [6, 13]

$$4U^2\left|\alpha\right|^6 + 4\Delta U\left|\alpha\right|^4 + \left[\left(\frac{\gamma}{2}\right)^2 + \Delta^2\right]\left|\alpha\right|^2 - \left|\xi\right|^2 = 0. \tag{7}$$

Depending on the specific parameter settings, either one, two, or three solutions of $|\alpha|^2$ are allowed in different parameter regimes. This observation is in stark contrast to the quantum-mechanical analysis, where a single unique SS solution is predicted throughout the entire parameter space. The stability of the system can be verified by checking whether $\partial\left|\xi\right|^2/\partial|\alpha|^2 > 0$ [6, 13]. At the boundaries of the hysteretic regime, we have

$$\left|\alpha\right|^2 = \frac{-2\Delta \pm \sqrt{\Delta^2 - 3\left(\gamma/2\right)^2}}{6U}, \tag{8}$$

which, in combination with Supplementary Eq. (7), can be used to draw the two boundaries of the hysteretic regime in the ξ-Δ space, as shown in Fig. 1b of the main text. It indicates that the hysteresis and bistability exist only in the regime $\Delta^2 > 3\,(\gamma/2)^2$, where the system can have a double-well potential. Outside this parameter regime, the potential has only a single minimum and there exists only a single unique SS solution. Depending on whether the driving strength is smaller or larger than either of the two boundaries, the single potential well is approximately localized at one of the two minima of the double-well potential. This observation leads to the method of initial state preparation, as will be discussed in detail in Supplementary Note 3.

Supplementary Note 2. SYSTEM CHARACTERIZATION

A. Experimental setup

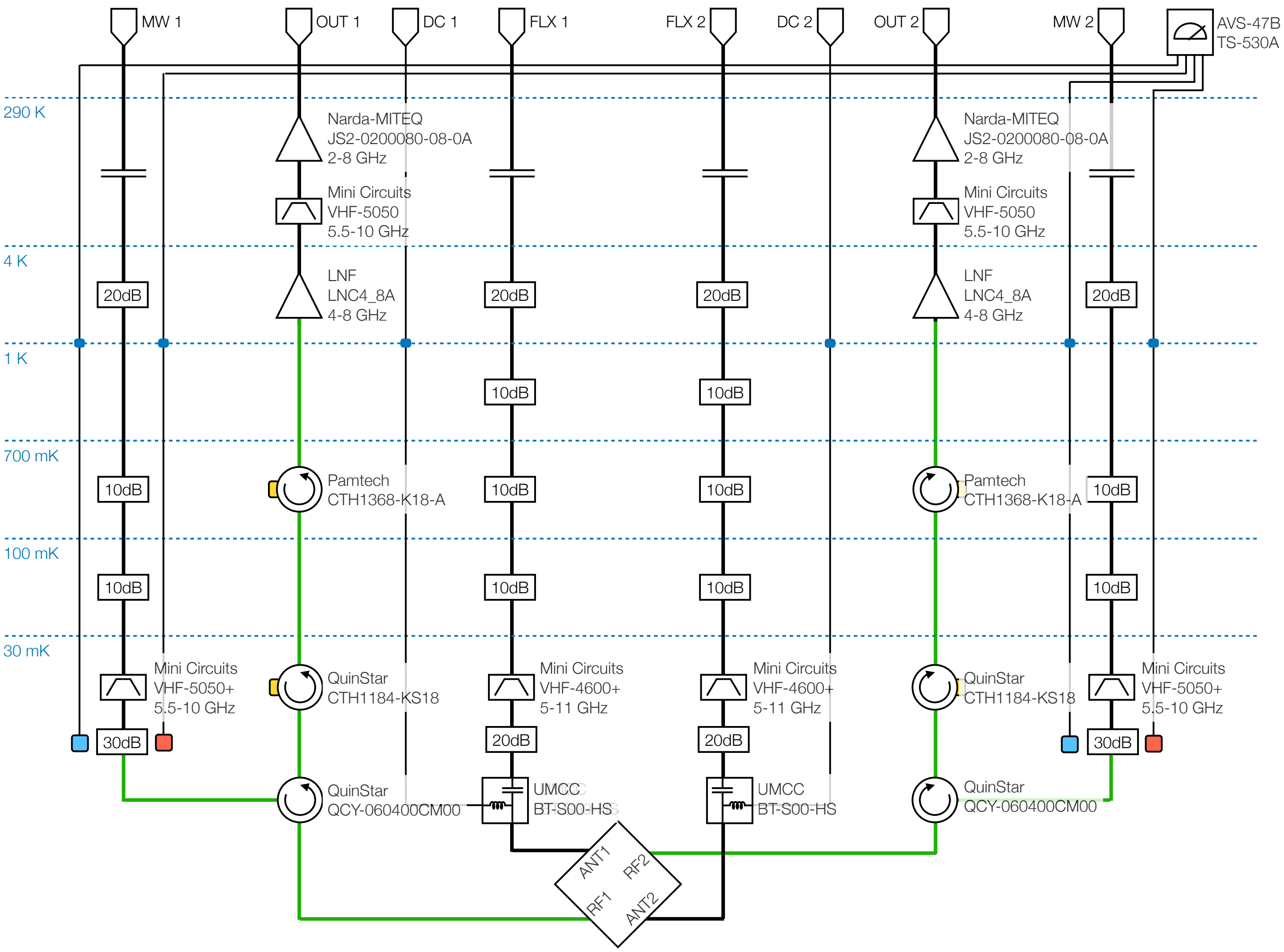

Supplementary Fig. 2: Schematic of the experimental setup. The sample is placed at the mixing chamber stage of a homemade wet-type dilution refrigerator, to which four microwave coaxial cables are connected. We also anchor two pairs of homemade cryogenic thermometers (blue box) and heaters (red box) to the two 30 dB attenuators at the base temperature to realize active control of the local temperature. Here, the blue dashed lines indicate the temperature stages of the cryostat, and the blue dots indicate a heat exchanger for cooling the DC wires. The green thick lines indicate NbTi cables.

The schematic of the experimental setup that is placed inside, or integrated on top of the dilution refrigerator is shown in Supplementary Fig. 2. From the top to the bottom, the temperature decreases from approximately 290 K, which is stabilized by using the Peltier cooler (Laird Hi-Pot tested 750VOC) and the temperature stabilizer (Telemeter TR12-PI-2Q2), to a minimum value of 30 mK at the sample stage. The input and output microwave lines, labelled as

MW 1/2, FLX 1/2, and OUT 1/2, are coupled to the two nonlinear resonators through the on-chip finger capacitors and the T-shaped antennae, as shown in Supplementary Fig. 1. Here, the microwave fields in MW 1/2 and OUT 1/2 are separated by using the cryogenic circulators (QuinStar QCY-060400CM00). We add also a 5.5-10 GHz high-pass filter in each of the input paths to isolate the sample from higher-frequency harmonics of the driving fields. In each of the the output paths, we add two circulators (QuinStar CTH1184-KS18, Pamtech CTH1368-K18-A) at 30 mK and 700 mK, respectively, to isolate the sample from the high-temperature thermal radiations and the possible back propagating fields coming from the HEMT amplifiers (LNC4_8A). At the top of the cryostat, we place a 5.5-10 GHz high-pass filter and amplify the cryogenic signal by a low-noise room-temperature amplifier (MITEQ JS2-0200080-08-0A) in each of the output line. These amplifiers are tightly integrated with the Peltier cooler, such that they operate at a stable temperature of around 17 °C. We place all of the described microwave components in an electromagnetically shielded room, while the temperature of the entire laboratory is stabilized around 27 °C by using the air conditioner.

For the DC part, we combine the output of the DC current sources (ADCMT 6241A) with the microwave fields in FLX 1/2 by using a bias-tee (UMCC BT-S00-HS), which are further connected to the T-shaped antennae on chip. In addition, two pairs of homemade cryogenic thermometers and heaters are clamped tightly to the two 30 dB attenuators at base temperature. They are connected to the AC resistance bridge (Picowatt AVS-47B) and the corresponding PID temperature controller (Picowatt TS-530A), in order to control the local temperatures of the two attenuators and generate blackbody radiation. This configuration is used to characterize the amplification gain and the noise temperature of the output paths, as will be discussed in Supplementary Note 2 E.

B. Control and readout modules

The schematic of the room-temperature setup for control and measurement is shown in Supplementary Fig. 3, which consists of three modules. The pulse-shaping module is designed to control the initial state of the nonlinear resonator and also to drive the system. We use a microwave signal generator (R&S SMF100A) to generate the radio frequency (RF) carrier wave. The field envelope is modulated by a double balanced mixer (Marki M1-0218LA) with its local oscillator (LO) port connected to the carrier wave and the intermediate frequency (IF) port to the first channel of an AFG (arbitrary function generator, Tektronix AFG3252). The RF port of the mixer is connected to the switch module for further signal routing. The second channel of the AFG is synchronized with the first one, which is used to trigger the measurement process of the ADC (analogue-to-digital converter, NI FlexRIO 5782). In addition, we place several attenuators, circulators, and filters in the configuration for the compatibility of different microwave devices.

The measurement module is designed to down-convert the RF signal to an IF frequency of $f_{\mathrm{IF}} = 62.5\,\mathrm{MHz}$ for pulsed heterodyne measurements. This choice of frequency avoids the possible beating between the signal and the higher order harmonics of the 10 MHz Rb frequency standard (SRS FS725), which synchronizes all the instruments in the lab. We use image rejection mixers (Polyphase IRM4080B) in the first two lines, OUT 1/2, to achieve a better signal-to-noise ratio (SNR), while a double balanced mixer (Marki M1-0218LA) is used in the third reference line for its relatively low price. However, we use the same LO field, which is generated by the microwave signal generator (R&S SMB100A), to drive all the three mixers for reaching a phase alignment. We also amplify the two channels, OUT 1/2, by low-noise room-temperature amplifiers (MITEQ AU1447R), and place several attenuators, filters, isolators, power dividers to improve the SNR. We note that the isolator (MCLI IS-19-1) is designed for the 4-8 GHz range, while it still works in the megahertz regime for our needs of preventing the possible back propagating fields from the IF amplifier.

Besides, we use several microwave coaxial switches (Agilent N1810TL, N1812UL) in the switch module to control the connectivity of different signal paths for different experimental purposes. The switches are controlled by a commercial controller (Agilent L4445A) with a homemade remote-control panel. For typical characterization experiments, where only the scattering coefficients are measured, we connect the two input ports, MW 1/2, and the two output ports, OUT 1/2, to the four channels of the VNA (vector network analyzer, Keysight PNA N5222A). However, for quadrature measurements we connect OUT 2 and the reference driving field to the two channels of the ADC, which has a sampling frequency of $f_{\mathrm{S}} = 250\,\mathrm{MHz}$. The driving field is connected to MW 2 for reflection-type measurements, or FLX 2 for transmission-type measurements.

C. Closed-system parameters

To determined the closed-system parameters, such as the resonant frequency, ω_{A}, and nonlinearity, U, we slowly sweep the current in either of the two antennae and measure the scattering coefficients. Here, we assume a linear

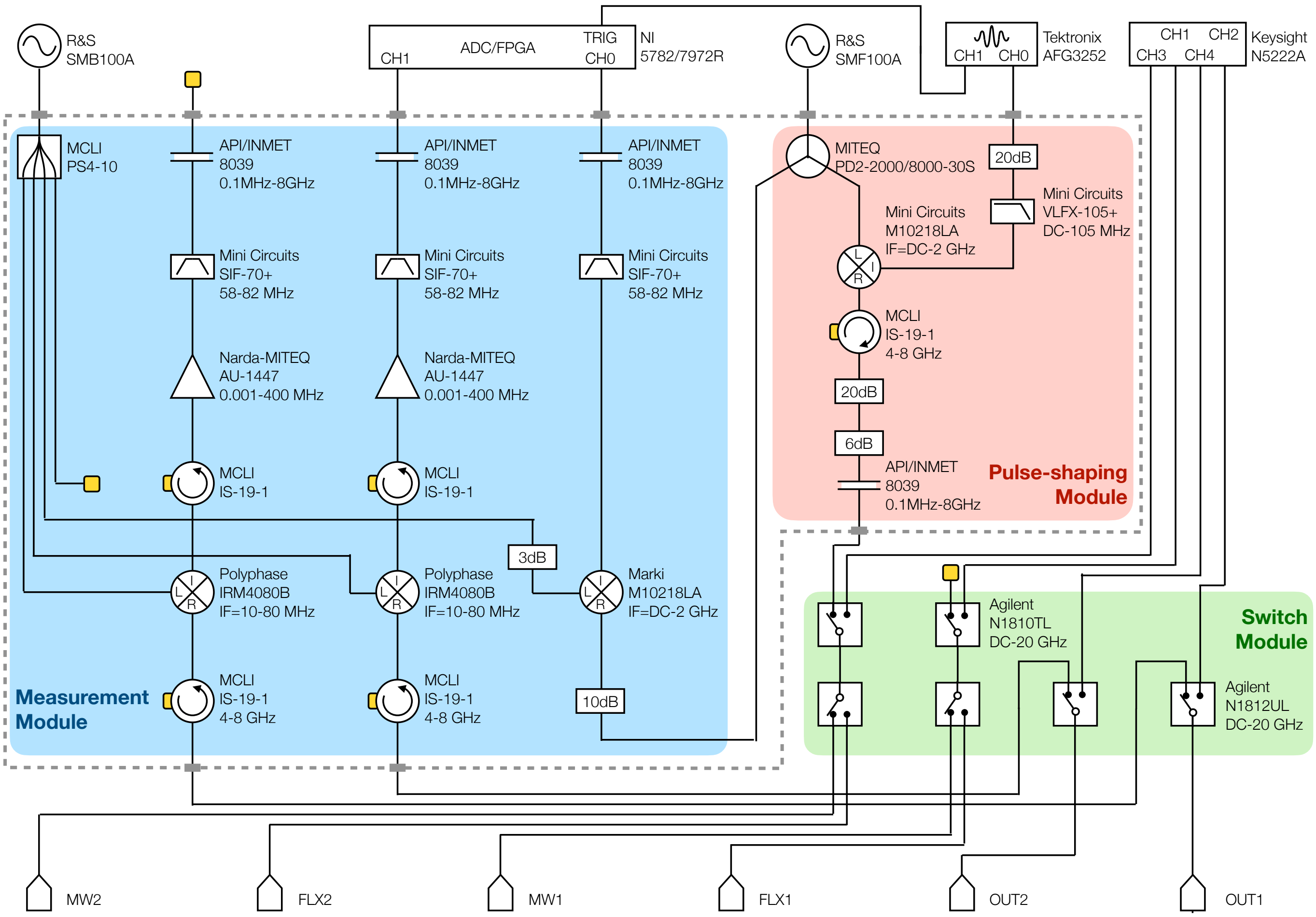

Supplementary Fig. 3: Schematic of the control and readout modules. The setup consists of three modules: The measurement module (blue), the pulse-shaping module (red), and the switch module (green). The components enclosed by the dashed lines are sealed in a $48 \times 24 \times 12\,\mathrm{cm}^2$ homemade copper box for electromagnetic shielding, grounding, and passive cooling. The switch module is placed outside the box to avoid potential stray magnetic field that may influence the other microwave devices.

relation between the flux and the applied currents [2]

$$\begin{pmatrix} \phi_1 \\ \phi_2 \end{pmatrix} = \begin{pmatrix} A_{11} & A_{12} \\ A_{21} & A_{22} \end{pmatrix} \begin{pmatrix} I_1 \\ I_2 \end{pmatrix} + \begin{pmatrix} \phi_{1,\mathrm{off}} \\ \phi_{2,\mathrm{off}} \end{pmatrix}, \tag{9}$$

where A is the crosstalk matrix, $\phi_{1,\mathrm{off}}$ and $\phi_{2,\mathrm{off}}$ are the offset flux threading into the two SQUID loops, and $\phi_0 = \hbar/2e$ is reduced flux quantum. We sweep respectively the two antenna currents from $-600\,\mu$A to $600\,\mu$A with 80 intermediate steps, and measure the scattering responses of the system by using the VNA. The sweeping speed is set to $1\,\mu$A/s, and the IF bandwidth of the VNA is set to 1 kHz. We average each data point over 100 times in a point-average mode. In principle, the output power of VNA should be set as low as possible in order to minimize the nonlinear effects [14]. However, considering the practical compromise between the SNR and the measurement time, we set the output power to 0 dBm with an additional 30 dB attenuation at room temperature. The exact attenuation of the input cables will be characterized in Supplementary Note 2 F. This configuration corresponds to an approximately 10 h measurement time for each characterization.

After getting the measurement results, we use an optimization method to find the best estimation of the 9 closed-system parameters of the sample, as well as the 6 parameters defined in Supplementary Eq. (9) for controlling the external flux. The automated fitting procedure not only provides the possibility to find the optimal estimation of the sample parameters, not matter local or global, in the huge parameter space, but also avoids possible biases in manual characterization and keeps a relatively objective criteria among different experiments. The characterization results are summarized in Supplementary Table 1, which has been reported in Supplementary Ref. 2. The slight difference of

CPW resonator	
length L	7.395×10^{-3} m
inductance per meter l	4.598×10^{-7} H
capacitance per meter c	1.697×10^{-10} F
SQUID #1, #2	
critical current I_{J1}, I_{J2}	1.566×10^{-6} A, 1.416×10^{-6} A
shunting capacitance C_{J1}, C_{J2}	9.394×10^{-16} F, 1.168×10^{-15} F
asymmetry d_{J1}, d_{J2}	2.136×10^{-1}, 1.937×10^{-1}
Antenna #1, #2	
flux offset $\phi_{1,\mathrm{off}}$, $\phi_{2,\mathrm{off}}$	$-3.902 \times 10^{-1}\,\phi_0$, $-1.149 \times 10^{-1}\,\phi_0$
flux change per current $d\phi_1/dI_1$, $d\phi_2/dI_1$	$6.088 \times 10^{-4}\,\phi_0/\mathrm{A}$, $9.927 \times 10^{-4}\,\phi_0/\mathrm{A}$
flux change per current $d\phi_1/dI_2$, $d\phi_2/dI_2$	$-3.715 \times 10^{-5}\,\phi_0/\mathrm{A}$, $-5.054 \times 10^{-4}\,\phi_0/\mathrm{A}$

Supplementary Table 1: Experimentally determined closed-system parameters of the system. Source data are provided as a Source Data file.

several parameters in Supplementary Table 1 and Supplementary Ref. 2 is attributed to the drift of sample parameters in different cool down.

D. Open-system parameters

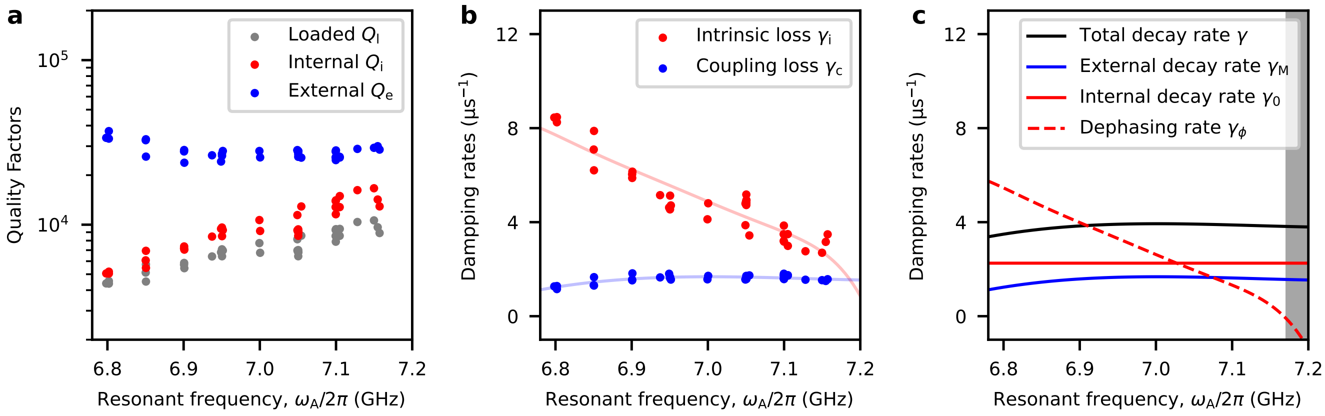

Supplementary Fig. 4: Experimentally determined open-system parameters of the second resonator. (a) We tune the resonant frequency to different values and characterize the internal, external, and loaded quality factors. **(b)** The result is transformed into the internal and external loss rates, γ_i and γ_c, respectively. **(c)** We separate the frequency-dependent and independent parts of γ_i into the dephasing rate, γ_ϕ, and the energy decay rate, γ_0, respectively. The latter can be further split into γ_A and γ_F. The shaded area indicates the frequency range not accessible by our sample. Source data are provided as a Source Data file.

Using an automated sample tuning procedure, we tune the second resonator to different frequencies and measure the reflection coefficient for characterizing the open-system parameters, such as the total energy dissipation rate, γ. The measurement data is processed by the recipe described in Supplementary Ref. 3, where the experimental imperfections, such as acquisition noise and circuit asymmetries, are corrected automatically. We note that the reflection coefficient of the effective one-resonator system is slightly different from a typical necklace-type $\lambda/2$ resonator, because we consider only the input and output fields at one single end of the resonator. In other words, we attribute all the photon-loss mechanisms, which include the resonator intrinsic loss as well as the photon loss through the flux line and the resonator-resonator coupling capacitor, into the internal quality factor, Q_i. Considering also the practical

distortions of the spectrum, we write [2, 3]

$$S_{22}(\omega) \approx Ae^{-j(\tau\omega+\varphi)}\left(1 - \frac{e^{j\phi}2Q_l/|Q_c|}{1+2jQ_l(\omega/\omega_r - 1)}\right), \tag{10}$$

Here, $1/Q_l = 1/Q_i + 1/Q_c$, and we have defined the reflection coefficient of the second resonator as S_{22}. To minimize the influence of the resonator nonlinearity and obtain a faithful characterization, we keep P_{in} sufficiently small. We set the power at the VNA output to -30 dBm and add $30 - 50$ dB room-temperature attenuations depending on the SNR at different frequencies. In this way, the spectrum is kept approximately Lorentzian such that the contribution of nonlinearity to the scattering coefficient can be fairly neglected [14].

Supplementary Fig. 4 summarizes the characterization results of the second resonator in the range between 6.80 and 7.20 GHz. Although the external Q factor is approximately a constant for different ω_A, the internal quality factor decreases when decreasing the resonant frequency. We note that similar observations are also reported in the literature [15, 16]. We attribute the change of Q_i to the possible effect of dephasing, which originates from the jitter of the resonant frequency due to flux noise. The flux noise can perturb the resonant frequency in time, such that the dephasing rate should depend on the derivative, $\gamma_\phi(\omega_A) = \eta d\omega_A/d\phi_{ex}$, of which the exact formula can be derived from the effective Josephson energy. Here, ϕ_{ex} is the flux bias and η is a constant to be determined. This interpretation is consistent with our observation, because $d\omega_A/d\phi_{ex}$ is increasing with decreasing frequency. By comparison, the external quality factor does not depend on the resonant frequency, which is also consistent with our observation. In these regards, we separate the energy dissipation and dephasing rates from the measured internal loss rate as $\gamma_i(\omega_A) = \gamma_0 + \gamma_\phi(\omega_A)$. The measured results fit very well with these relations, which indicates a good understanding of the dissipation mechanisms of our system. Besides, we also use a third-order polynomial to fit the weak dependance of the external decay rate on the frequency, $\gamma_M(\omega_A) \equiv \gamma_c(\omega_A)$, which may originate from possible experimental imperfections. In total, we obtain the total energy dissipation rate $\gamma(\omega_A) = \gamma_0 + \gamma_M(\omega_A)$ and the dephasing rate $\gamma_\phi(\omega_A)$. The characterization result shows that the second resonator is under coupled with $\gamma_0 = 2.26\,\mu s^{-1}$ and $\gamma_M = 1.59\,\mu s^{-1}$ on average. The total energy dissipation rate, γ, dominates the dephasing rate, γ_ϕ, for $\omega_A/2\pi \geq 6.9$ GHz. This indicates that the dephasing effect may be fairly neglected in this frequency range. We note that γ_0 is a combination of the intrinsic damping rate, γ_A, and the coupling induced damping rate, γ_F, as discussed in Supplementary Note 1 A. With no knowledge on the ratio between the two rates, we simply assume that $\gamma_A = \gamma_F = \gamma_0/2$ in the rest of the discussions, which already shows a good consistency between the simulation and our experimental results.

E. Gain and noise in the output path

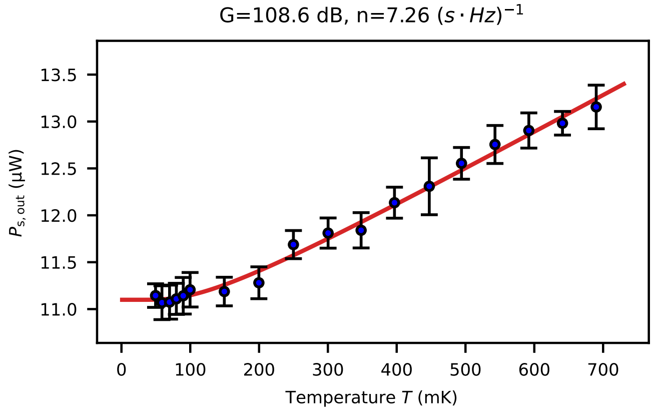

Supplementary Fig. 5: Experimentally determined parameters of the output channel, OUT 2. We tune the second resonator to approximately 7.10 GHz and measure the blackbody radiation from the signal path, OUT 2, within a ± 2 MHz bandwidth around the central frequency 6.95 GHz (blue dots). The blackbody radiation is generated by a 30 dB heatable attenuator which is mounted just at the sample input. The error bars represent the standard deviation among 16 independent experiments, and the red solid curve is the numerical fit. Source data are provided as a Source Data file.

We relate the output signal field at the cryogenic temperature, $b_{s,in}$, and the fields to be measured at the room

temperature, $b_{\rm s,out}$, by the Caves formula [17–19]

$$b_{\rm s,out} \approx \sqrt{G}\left(b_{\rm s,in} + b^{\dagger}_{\rm n,in}\right), \tag{11}$$

where $b_{\rm n,in}$ is the field operator of the amplification noise, and G is the power gain of the amplification chain. Here, we have neglected the difference between G and $(G-1)$ for a sufficiently large gain ($G \gg 1$), which is valid in common experiments of superconducting quantum circuits. We use the thermal noise as a resource to obtain a precise knowledge of G and $b_{\rm n,in}$ [20–27].

The Planck's law describes the energy density of a field emitted by a blackbody thermalized at temperature T. A straightforward derivation of Planck's law can be obtained by recalling the properties of a single-mode thermal state at temperature T, where the average photon number is $\bar{n}_T(\omega) = 1/\left\{\exp\left[\hbar\omega/(k_{\rm B}T)\right] - 1\right\}$. Here, $\bar{n}_T(\omega)$ has the dimension of photon number per second per bandwidth. Straightforwardly, the power of thermal radiation in a narrow band, $2B/2\pi$, can be obtained as $P = B\hbar\omega\bar{n}_T(\omega)/\pi$. The value of P can be calculated from the measured I/Q quadratures, that is $P = \left(\overline{I^2} + \overline{Q^2}\right)/(2Z_0)$. Here, we have assumed a perfect impedance match at the ADC input with $Z_0 = 50\Omega$. The factor of 2 originates from the sinusoidal nature of the microwave field.

To characterize the parameters G and $\langle b^{\dagger}_{\rm n,in} b_{\rm n,in}\rangle$, we tightly clamp a homemade cryogenic heater and a homemade cryogenic thermometer to a 30 dB attenuator to generate the blackbody radiation at the sample input (See Supplementary Note 2 A for detail). The heater is a 100 Ω resistor (Vishay MCT 0603), of which the temperature, T, is measured and controlled by the AC resistance bridge (Picowatt AVS-47B) and the PID temperature controller (Picowatt TS-530A). The 30 dB attenuator can be modeled as a beam splitter which transmits 0.1% of its input signal and 99.9% of the thermal radiation from the environment at temperature T. Assuming that the measurement bandwidth is largely detuned from the resonant frequency of the resonator, $\omega_{\rm A}$, this blackbody radiation can be fully reflected at the sample input, and then amplified and measured as a finite power $P_{\rm out} \equiv B\hbar\omega_{\rm A}\langle b^{\dagger}_{\rm s,out} b_{\rm s,out}\rangle/\pi$. In the form of the Caves formula, we have

$$P_{\rm s,out} \approx \frac{GB\hbar\omega_{\rm A}}{\pi}\left[\bar{n}_T(\omega_{\rm A}) + n + 1\right]. \tag{12}$$

Here, we have defined $n \equiv \langle b^{\dagger}_{\rm n,in} b_{\rm n,in}\rangle$, and the constant 1 comes from the commutation relation, $b^{\dagger}_{\rm n,in} b_{\rm n,in} = b_{\rm n,in} b^{\dagger}_{\rm n,in} - 1$. In our experiment, we calibrate G and n by sweeping the temperature T.

Supplementary Fig. 5 shows the relation between the measured power, $P_{\rm s,out}$, and the temperature, T, for the output channel OUT 2. The resonant frequency of the second resonator is tuned to approximately 7.10 GHz, while we measure the microwave signal at 6.95 GHz within a ±2 MHz bandwidth. We note that $B/2\pi = 2$ MHz is the cut-off frequency of the low-pass digital filter on FPGA, while the two sidebands of the microwave signal around 6.95 GHz are folded into a single sideband during the digital down conversion process. The local temperature of the heatable attenuator is varied from approximately 50 to 700 mK with a precision of ±2.5 mK during the measurement time. At each temperature, we average the measured signal power by approximately 5×10^4 times, where the error bars are obtained by repeating this procedure 16 times. We use the least square estimation method to fit Supplementary Eq. (12) with the measurement averages. We observe a power gain of $G = 109$ dB for the output path, with the mean noise photon number of $n = 7.3\,(\rm s \cdot Hz)^{-1}$ corresponding to a noise temperature of 2.4 K.

F. Attenuation and offset in the input path

Having the knowledge of the gain, G, and noise photon number, n, in the output path, OUT 2, we move on to characterize the attenuation, A, of the input path, MW 2. Assuming that the power of a signal generator is set as $P_{\rm d}$, we relate the measured signal power, $P_{\rm s,out}$, and $P_{\rm d}$ in a similar form of the Caves formula

$$P_{\rm s,out} \approx G\left[AP_{\rm d} + \frac{B\hbar\omega_{\rm A}}{\pi}(n+1)\right]. \tag{13}$$

The aim of determining A is to establish a relation between $P_{\rm d}$ and the driving strength, ξ, in the system Hamiltonian, that is [28]

$$\xi = -i\sqrt{\gamma_{\rm M/F} A P_{\rm d}/\hbar\omega_{\rm d}}. \tag{14}$$

Here, we have assumed the driving field to be in a coherent state. In our experiment, we drive the system via a homemade pulse-shaping module, where the carrier wave generated by the signal generator (R&S SMF100A) is

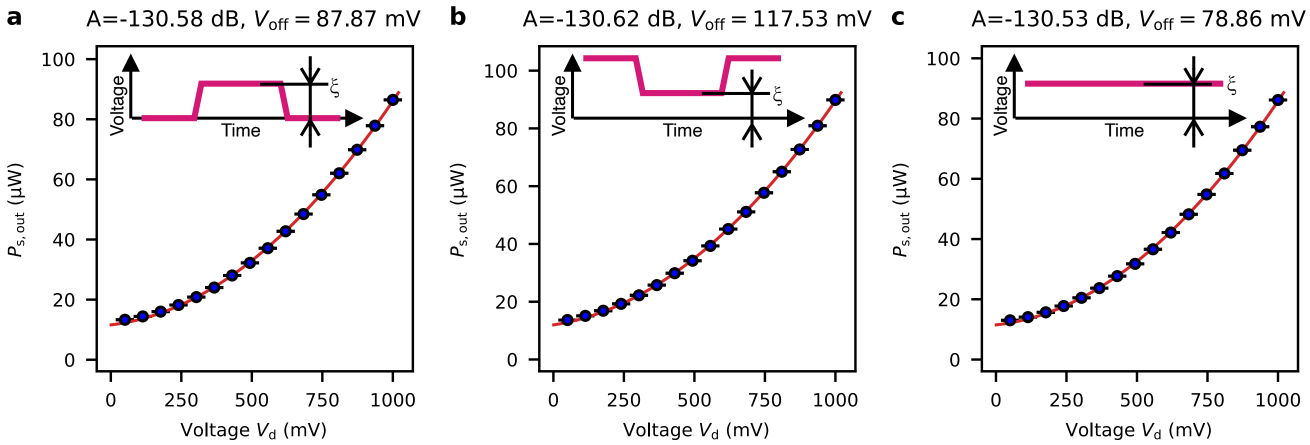

Supplementary Fig. 6: Experimentally determined parameters of the input channel, MW 2. We drive the system through the input path, MW 2, and measure the reflected signal from the path, OUT 2, within ±2 MHz around the central frequency 7.00 GHz (blue dots). The error bars represent the standard deviation among 16 independent experiments, which are smaller than the size of the dots, and the red solid curve is the numerical fit. Panels (a)-(c) correspond to different pulse shapes. Source data are provided as a Source Data file.

modulated by a voltage signal, $V_{\rm d}$, generated by the AFG (Tektronix AFG3252), as described in Supplementary Note 2 B. We assume a simple relation between $P_{\rm d}$ and the pulse amplitude $V_{\rm d}$ as $P_{\rm d} = \left(V_{\rm d} - V_{\rm off}\right)^2/(2Z_0)$. Here, $V_{\rm off}$ is the offset voltage in the setup, which originates from the imperfect grounding of mixers in the pulse-shaping module. Besides, we assume a perfect impedance match with $Z_0 = 50\,\Omega$. The goal of the input characterization experiment is to determine the values of A and $V_{\rm off}$.

Supplementary Fig. 6 shows the measured signal power, $P_{\rm out}$, as a function of the pulse amplitude, $V_{\rm d}$, for the input channel, MW 2. Here, we set the carrier frequency of the input field to 7.00 GHz and vary the pulse amplitude from 50 mV to 1000 mV. The other parameters are set to be exactly the same as for the output characterization experiments. We employ three different pulse shapes for characterization. Correspondingly, the characterized attenuations are $A = -130.6$ dB, -130.6 dB, and -130.5 dB, respectively, which are almost identical to each other. However, the offset voltage shows a clear dependance on the pulse shape. The results are $V_{\rm off} = 88$ mV, 118 mV, and 79 mV, which vary by approximately 50 mV for the three different pulse shapes shown in Supplementary Fig. 6. Besides the imperfect grounding, this may also be attributed to the finite on/off ratio of the mixer, which mixes the carrier wave with the voltage signal in the pulse-shaping module. However, we note that a 50 mV offset voltage corresponds to an inaccuracy of $\xi/2\pi$ being less than 5 kHz, which is negligibly small in all of our experiments.

Supplementary Note 3. EXPERIMENTAL METHODS

The pulsed measurement plays a fundamental role in revealing the non-equilibrium quantum dynamics of the Duffing oscillator, as is schematically shown in Supplementary Fig. 7. The basic logic of the experiment is (i) to prepare the system in one of the two wells, (ii) to drive the system at ξ, (iii) to wait for a controllable time τ, and (iv) to start a short measurement. We note that the control parameters are not swept in a continuous fashion, which is different from the relevant experiments in the literature.

A. Initial state preparation

To prepare the system in different wells at the initial time, we set the driving strength at either zero (pulse-A) or the maximum value one can achieve (pulse-C), which is limited by approximately 4.7 V at the AFG output when using the pulse-generation mode. As discussed in Supplementary Note 1 C, the system has a single-well potential at a sufficiently small or large driving strength, which corresponds to one of the two potential wells, respectively. We wait for approximately 4 μs to let the system reach the SS of the single-well potential, which is more than 10 times larger than the free relaxation time, $1/\gamma$. Next, we switch the driving strength to ξ, which defines the driving strength in Supplementary Eqs. (3) and (6). The switching time is usually set to 250 ns, which aims to provide a smooth but

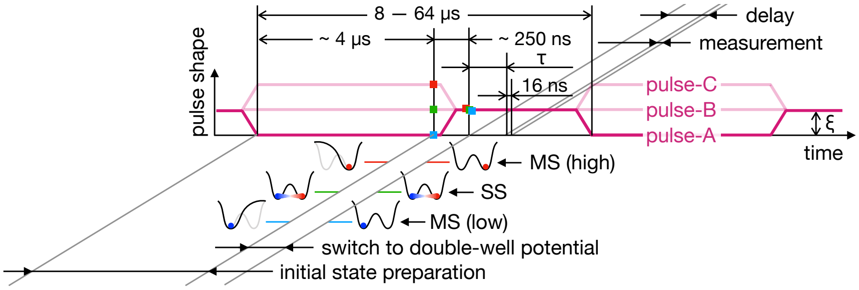

Supplementary Fig. 7: The schematic of the pulsed heterodyne measurement protocol. We prepare the initial state of system in one of the two potential wells by driving it with either a zero-amplitude (pulse-A) or a high-intensity field (pulse-C). Then, the driving strength is switched to ξ and lasts for a controllable time τ before we perform a 16 ns quadrature measurement. This procedure is repeated for more than 10^6 times to accumulate a histogram of the field quadratures. In certain experiments, we also drive the system with a constant driving field with driving strength ξ (pulse-B), which prepares the system in the SS at the initial time.

relatively fast transition between the initial and final values. Depending on the exact value of ξ and Δ, the system can have a double-well potential in the so-called hysteretic regime, while the initial state is prepared in either of the two wells. We also drive the system with a constant driving field (pulse-B), where the system is initially prepared in the SS.

B. Pulsed heterodyne measurement

We always wait for a time duration of τ before starting a measurement. In order to capture the non-equilibrium dynamics of the system, we measure only one period of the IF signal, which is 16 ns. Here, only 4 data points are recorded in a single measurement event, corresponding to one data point of the field quadratures, $I + iQ$, with a time resolution of 16 ns. Then, we initialize the system and repeat the same measurement procedure by 10^6 – 10^9 times depending on the required measurement accuracy, each of which is triggered at the same relatively time after the initialization. Because the experimental conditions are kept the same, the measurement results should also be the same within the uncertainty range defined by the quantum fluctuations. One can figuratively understand the pulsed measurement as using millions of ADCs that measure the system at the same time. Then, we concatenate the data recorded by the different virtual ADCs into a long trace and apply a low-pass filter to increase the SNR. Because the resulting signal is not sequenced in real time, the cut-off frequency, or the ring-up time, of the filter does not influence the time resolution of the measurement result. It indicates that one can apply a relatively narrow-band digital low-pass filter (~ 2 MHz in this case) to improve the SNR but keep the 16 ns time resolution of the result.

C. Photon correlations

During the pulsed measurement, we record the two signal moments, $\langle b_{\rm s,out}\rangle$ and $\langle b^\dagger_{\rm s,out} b_{\rm s,out}\rangle$, as well as the histogram of $b_{\rm s,out}$ in a 128 × 128-dimentional matrix. The measured histogram is the Q function of the output field, $b_{\rm s,out}$, which is a convolution between the input field, $b_{\rm s,in}$, and the noise field, $b_{\rm n,in}$, [29–31]

$$Q_{\rm s,out}(\gamma,\gamma^*) = \frac{1}{G-1}\int d\alpha^2 Q_{\rm s,in}(\alpha,\alpha^*) P_{\rm n,in}\left(\frac{\gamma^* - \sqrt{G}\alpha^*}{\sqrt{G-1}}, \frac{\gamma - \sqrt{G}\alpha}{\sqrt{G-1}}\right), \tag{15}$$

where $Q_{\rm s,out}$, $Q_{\rm s,in}$, and $P_{\rm n,in}$ are the quasi-distribution functions of the three fields. Combining Supplementary Eq. (15) with the input-output relation, $b_{\rm s,in} = \sqrt{\gamma_{\rm M}}a$, one can calculate all orders of the signal moments as

$$\langle b^{\dagger k}_{\rm s,out} b^l_{\rm s,out}\rangle = \left(\frac{\gamma_{\rm M} BG}{\pi}\right)^{\frac{k'+l'}{2}} \sum_{k'=0}^{k}\sum_{l'=0}^{l} C_k^{k'} C_l^{l'} \langle a^{\dagger k'} a^{l'}\rangle \langle b^{\dagger k-k'}_{\rm r,out} b^{l-l'}_{\rm r,out}\rangle, \tag{16}$$

where $\langle b^{\dagger k}_{\rm r,out} b^l_{\rm r,out}\rangle = (BG/\pi)^{\frac{k+l}{2}} \langle b^k_{\rm n,in} b^{\dagger l}_{\rm n,in}\rangle$ is measured when the resonator is in the vacuum state, $C_k^{k'}$ is the binomial coefficient. The filter bandwidth is typically set to $B/2\pi = 2$ MHz. However, because the coupling strength between

the resonator and the microwave line, MW 2, is approximately 2 times smaller than $2B$ (See Supplementary Note 2 D), we compensate the power gain in our analysis code by -3 dB to characterize the intra-resonator photon number. This correction leads to a good agreement between all of our experimental results and the simulations with no fitting parameter. In certain tasks where a high-precision estimation of the photon number is required, for example, the quantum state tomography discussed in Supplementary Note 4 C, we will fine tune the value of G in a ± 1 dB range.

Supplementary Note 4. SUPPLEMENTARY DATA

A. Closure of the hysteresis loop in the long-time limit

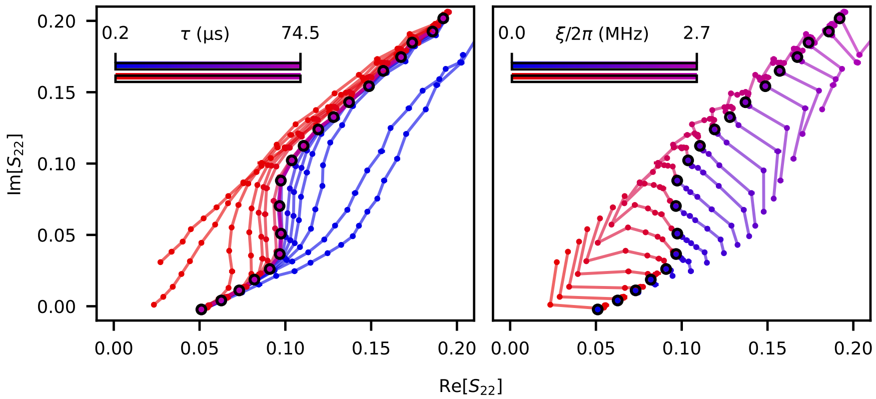

Supplementary Fig. 8: Power-delay sweep obtained by a reflection-type measurement. The reflection coefficients, S_{22}, corresponding to the two MSs branches (blue and red) form a closed loop, which converge to the unique SS solution (back circle) with increasing τ. Here, the error bars represent the standard deviation over 8 independent experiments. Source data are provided as a Source Data file.

One major difference between the classical and quantum theories of the Duffing oscillator is the number of SSs. The former predicts two in the hysteretic regime, which are localized in either of the two potential wells. However, the latter predicts one unique SS in the entire parameter space. In this regard, one straightforward way to verify the quantum dynamics of the Duffing oscillator is to prepare the system in either of the two wells and wait for a long time before measurement. In the absence of thermal noise, the two classical SSs remain in the well such that the area of the hysteresis loop should not decrease with τ. However, the loop area must decrease in the quantum perspective, because of the uniqueness of the SS. This latter prediction is confirmed in Fig. 2A of the main text. Moreover, the two branches must converge to a single curve corresponding to that of the SS when $\tau \gg 1/\min_\xi \delta_1(\xi)$, where $\delta_1(\xi)$ is the Liouvillian gap as a function of the driving strength, ξ. This phenomenon is not demonstrated in Fig. 2A of the main text, because τ is limited by $45\,\mu$s there and we did not measure the SS curve in that experiment. As a supplementary data, we plot in Supplementary Fig. 8 a similar measurement where τ goes up to $75\,\mu$s. In this case, the SS is also measured. Here, the closure of the hysteresis loop is observed at $\tau \simeq 55\,\mu$s, which is larger than 230 times of the free relaxation time $1/\gamma$. In addition, the two MS branches converge continuously to the SS curve. This result, in combination with Fig. 2A of the main text, demonstrates the uniqueness of the SS, as predicted in the quantum theory.

B. Extracting the Liouvillian gap from time-domain measurements

To extract the Liouvillian gap, δ_1, from the time-domain measurements, we calculate the distance between the two MSs branches for each ξ as a function of τ. Supplementary Fig. 9 shows the raw data of Fig. 2C of the main text with fitted results. Here, we did not correct the cable delay in the time axis, which is measured to be approximately $0.25\,\mu$s. For each ξ, we fit the data in the $\tau \geq 0.5\,\mu$s range with an exponential function. Because $\tau > 1/\gamma$, the fitted decay rate can be fairly regarded as the Liouvillian gap, which dominates the relaxation of the system in the long-time limit. As described in the main text, the fitted value of δ_1 is approximately equal to the energy dissipation rate, γ, at either low or high driving strengths. However, it decreases over two orders of magnitude when approaching to the

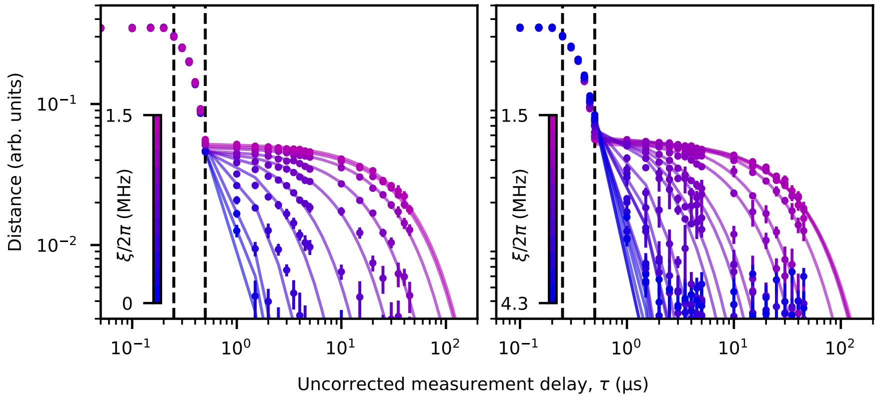

Supplementary Fig. 9: Power-delay sweep obtained by a reflection-type measurement. Shown are the raw data (dots) for extracting the Liouvillian gap, and the exponential fitting results (solid curves). With the increase of the driving strength in the $0 \leq \xi/2\pi \leq 1.5\,\text{MHz}$ regime, the relaxation process becomes increasingly slower. However, the relaxation becomes increasingly faster if we increase further the driving strength, $1.5\,\text{MHz} \leq \xi/2\pi \leq 4.3\,\text{MHz}$. In all the panels, the error bars represent the standard deviation over 16 independent experiments. Source data are provided as a Source Data file.

critical driving strength, $\xi^*/2\pi = 1.51\,\text{MHz}$. This result explains the two-stage relaxation process of the system, as shown in Fig. 2B of the main text.

C. Quantum state tomography of the phase transition process

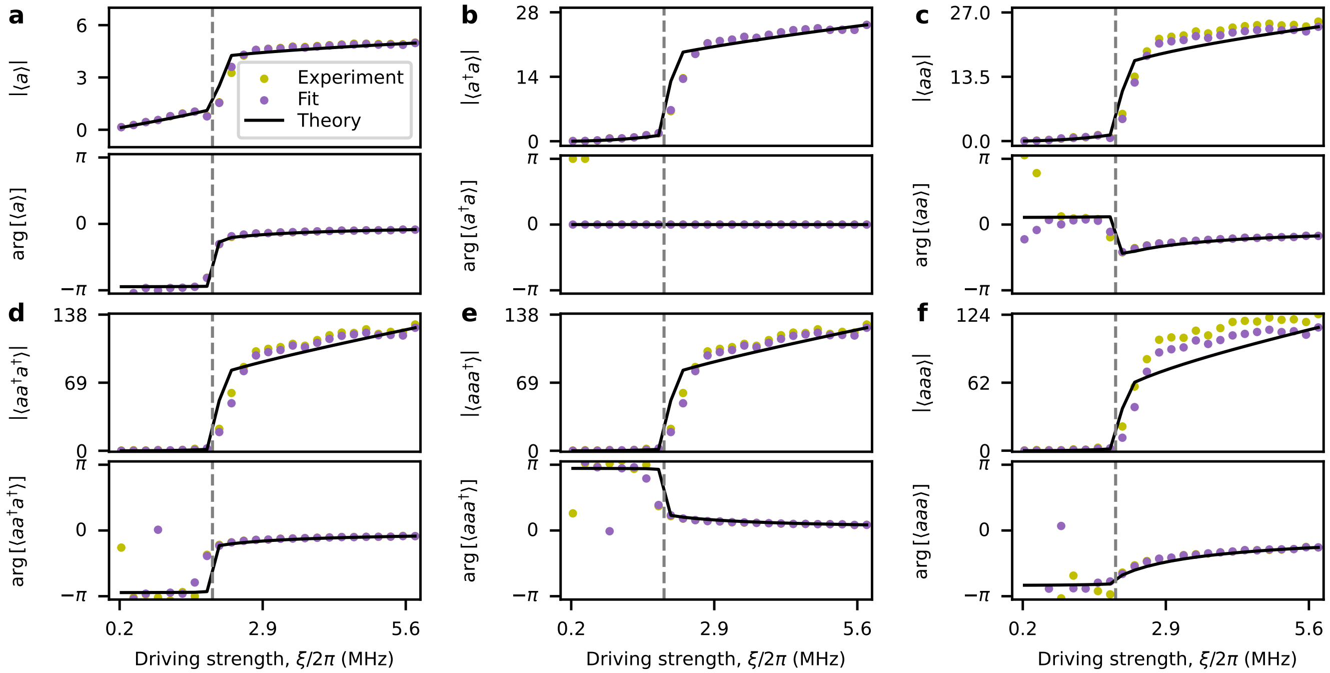

Supplementary Fig. 10: The first three orders of signal moments. The measured amplitude and phase of the signal moments show an excellent fit to the theoretical prediction in Supplementary Eq. (4) with no fitting parameter, which provides the opportunity for quantum state tomography. **(a)-(f)** correspond to the signal moments, $\langle a\rangle$, $\langle a^\dagger a\rangle$, $\langle a^2\rangle$, $\langle aa^{\dagger 2}\rangle$, $\langle a^2a^\dagger\rangle$, $\langle a^3\rangle$, respectively. The first two orders of moments, **(a)-(c)**, are used for quantum state tomography. Source data are provided as a Source Data file.

Quantum state tomography in our experiment is achieved by combining Supplementary Eqs. (4) and (5). On the one hand, the exact Wigner function can be fully determined by the two parameters, c and d, in Supplementary Eq. (5). On the other hand, these two parameters are closely related to the signal moments in Supplementary Eq. (4). We thus find the best fit of c and d from the first two orders of signal moments according to Supplementary Eq. (4), and insert the fitted values to Supplementary Eq. (5) to get the Wigner function. Because c is a complex number and d is real, one needs at least the information of the two moments, $\langle a\rangle$ and $\langle a^\dagger a\rangle$, to determine the two parameters. Here, we take also the $\langle a^2\rangle$ term into consideration, which makes the fitting problem overdetermined, and thus increases the reliability of the tomography result.

To minimize the influence of the dephasing effect, we only perform quantum state tomography at $\omega_\mathrm{A}/2\pi = 7.15\,\mathrm{GHz}$. Supplementary Fig. 10a-f compares the measured signal moments and the simulation results up to the third order, which shows a good agreement between theory with no fitting parameter and experiment. The shown excellent fit between theory and experiment thus justifies the feasibility of extracting the values of c and d from the measured signal moments for quantum state tomography. In that process, we fine tune the power gain for each order of the signal moments, as well as the global phase offset, according to the last data point. The tomography result, as shown in Fig. 4 of the main text, is obtained from the first 2 orders of signal moments shown in Supplementary Fig. 10a-c.

We comment that the described procedure relies on the priori knowledge of the SS and thus lacks general objectivity. Alternative methods, such as coupling a probe qubit to the resonator [32–35], may provide a more general tomography result. However, we note that the model itself does not necessarily predict the non-classical feature of the SS in the $0.57\,\mathrm{MHz} \le \xi_0/2\pi \le 0.71\,\mathrm{MHz}$ range, as shown in Fig. 4 of the main text. We see that we are able to reveal the transition process with a simple physical model but no fitting parameter, and also to obtain a consistent understanding between theory and experiment among independent experiments.

D. Squeezing levels in the two phases besides the phase transition

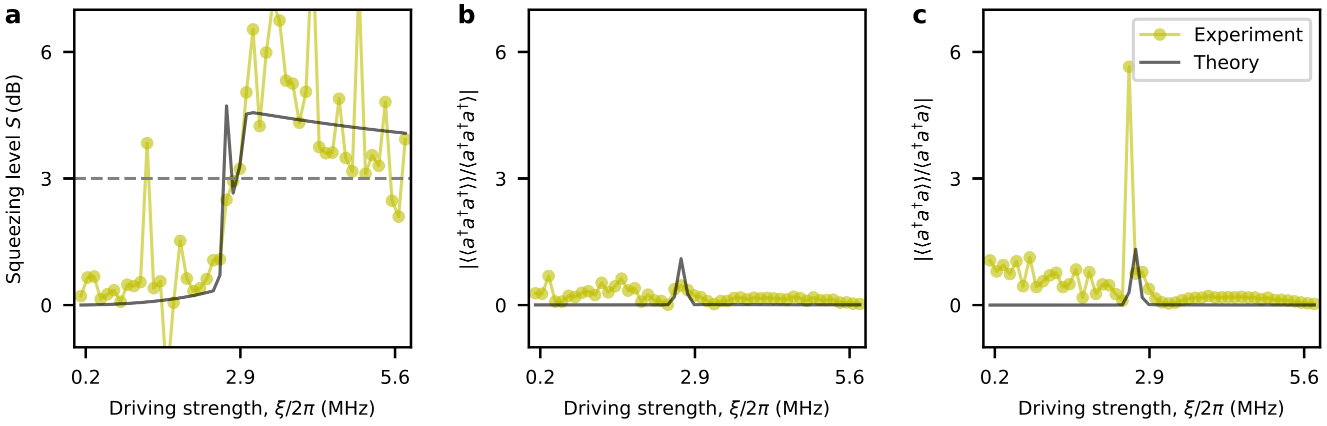

Supplementary Fig. 11: The squeezing level as a function of the driving strength. (a) The critical point, $\xi^*/2\pi = 2.76\,\mathrm{MHz}$, separates the system into two different phases with drastically different squeezing levels S. The value of S is approximately zero before the transition, which indicates a coherent phase of the system. After the transition, the squeezing level is approximately 3 dB, which corresponds to a squeezed phase. **(b)-(c)** The measured third-order cumulants, $\langle\langle a^{\dagger 3}\rangle\rangle$ and $\langle\langle a^{\dagger 2}a\rangle\rangle$, are close to zero except in the transition process. It demonstrates the feasibility of Gaussian-state approximation. Here, the resonant frequency is set as $\omega_\mathrm{A}/2\pi = 7.15\,\mathrm{GHz}$, and the detuning frequency is $\Delta/2\pi = 2.28\,\mathrm{MHz}$. Source data are provided as a Source Data file.

As shown in Fig. 4 of the main text, the SSs in the two phases are either a coherent or squeezed state outside the hysteretic regime. We use a Gaussian function to describe them and calculate the corresponding squeezing levels [36]. By definition, a Gaussian state is a rotated, squeezed, and displaced thermal state $\rho = D(\alpha)S(\zeta)R(\phi)\rho_T R^\dagger(\phi)S^\dagger(\zeta)D^\dagger(\alpha)$, where $D(\alpha)$, $S(\zeta)$, and $R(\phi)$ are the displacement, squeeze, and rotation operators. The squeezing level can be defined as $S = -20\,|\zeta|\log_{10}(e)$ where e is the exponential constant. On the other hand, for Gaussian states we have

$$\tanh(2\,|\zeta|) = \frac{\langle a^2\rangle - \langle a\rangle^2}{\langle a^\dagger a\rangle + 1/2 - |\langle a\rangle|^2}. \tag{17}$$

One can thus calculate the squeezing level of the two phases according to the measured signal moments: $\langle a\rangle$, $\langle a^\dagger a\rangle$, and $\langle a^2\rangle$. Supplementary Fig. 11 a shows the squeezing level of the system as a function of the driving strength. The critical point, $\xi^*/2\pi = 2.76\,\mathrm{MHz}$, separates the system into two different phases with drastically different squeezing levels. The value of S is approximately zero before the phase transition, but jumps to approximately 3 dB afterwards. This observation reveals the two distinct phases of the DPT with respect to the different squeezing levels.

We note that the Gaussian-state approximation breaks down around the critical point, where the SS is a mixture of the two phases [36]. This can be seen from the unexpected peak in the theory curve around ξ^*. To crosscheck the Gaussianity of the SS, we calculate the third order cumulants [37]

$$\langle\langle a^{\dagger 3}\rangle\rangle = \langle a^{\dagger 3}\rangle - 3\langle a^{\dagger 2}\rangle\langle a^\dagger\rangle + 2\langle a^\dagger\rangle^3, \tag{18}$$

$$\langle\langle a^{\dagger 2}a\rangle\rangle = \langle a^{\dagger 2}a\rangle - \langle a^{\dagger 2}\rangle\langle a\rangle - 2\langle a^\dagger a\rangle\langle a^\dagger\rangle + 2\langle a^\dagger\rangle^2\langle a\rangle. \tag{19}$$

These cumulants should be zero for Gaussian states. Supplementary Fig. 11 b-c shows the experimental result, where we have normalized the cumulants by the corresponding moments, $\langle a^{\dagger 3}\rangle$ and $\langle a^{\dagger 2}a\rangle$, to compensate the relatively large variance at high driving strength. We observe that the cumulants are close to zero outside the hysteretic regime, which justifies the Gaussian-state approximation. The seeming large deviation at low driving strengths is mainly caused by the small valued numerators, $\langle a^{\dagger 3}\rangle$ and $\langle a^{\dagger 2}a\rangle$, while the absolute mean of the cumulants, $\langle\langle a^{\dagger 3}\rangle\rangle$ and $\langle\langle a^{\dagger 2}a\rangle\rangle$, are merely 0.07 and 0.19 with standard deviation 0.20 and 0.16, respectively.

E. The dephasing effect and possible two-photon processes

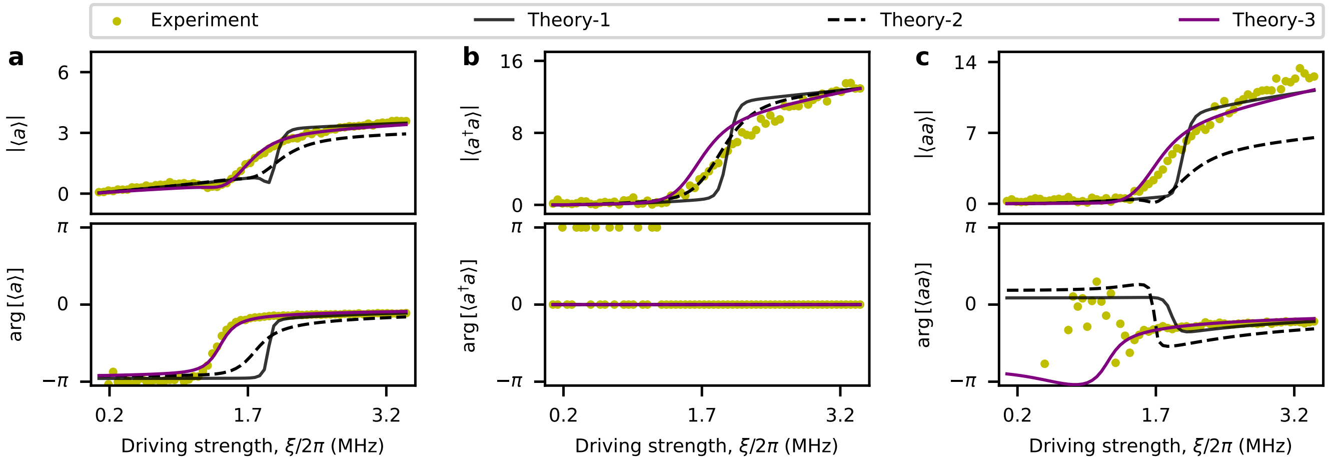

Supplementary Fig. 12: Comparison between experimental and numerical results for the first two orders of signal moments. The yellow dots represent the experimental results. The black solid curves show the analytical result in Supplementary Eq. (4), where only energy dissipation is considered (Theory-1). The dashed black line shows the master equation simulation with dephasing effect and a finite thermal photon number of the environment (Theory-2). It captures the the slower transition rate observed in experiment but leads to a large discrepancy in $\langle a^2\rangle$. We also consider a weak two-photon drive and loss process in the model (Theory-3, purple solid), which provides a good agreement with all the three signal moments. Source data are provided as a Source Data file.

So far, we have neglected dephasing effects in the discussion. This is feasible because the determined dephasing rate, γ_ϕ, is smaller than the energy dissipation rate, γ, in the frequency range of interest (See Supplementary Note 2 D for the characterization results). It is also justified by showing the excellent agreement between theory and experiment for all the first three orders of signal moments, as shown in Supplementary Fig. 10. However, this agreement exists only at high resonant frequencies. As can be seen in Fig. 3 of the main text, the experimentally observed transition curve is less steep than that predicted by the model at lower frequencies. As discussed in Supplementary Note 1 A, we attribute the reduced steepness to the presence of finite dephasing, since the dephasing rate increases when going to lower frequencies.

To achieve a quantitive understanding of the experimental data, we add the dephasing term by hand and move

further to the Schrödinger picture. The master equation in the Lindblad form reads

$$\partial_t\rho(t) = -i\left[H_{\text{eff}}, \rho(t)\right] + \frac{\gamma}{2}\left(n_{\text{T}}+1\right)\mathcal{D}\left[a\right]\rho(t) + \frac{\gamma}{2}n_{\text{T}}\mathcal{D}\left[a^\dagger\right]\rho(t) + \frac{\gamma_\phi}{2}\mathcal{D}\left[a^\dagger a\right]\rho(t). \tag{20}$$

Here, $\rho(t)$ is the density operator, H_{eff} is the effective Hamiltonian of the system, and $\mathcal{D}\left[a\right]$ and $\mathcal{D}\left[a^\dagger a\right]$ are the Lindbladian superoperators. Besides, we consider also a finite temperature of the bath n_T. The value of the energy relaxation and the dephasing rates, γ and γ_ϕ, have been determined in Supplementary Note 2 D.

Supplementary Fig. 12 compares the measured signal moments with the simulation results. Compared with the analytical result with $\gamma_\phi = 0$, a finite dephasing rate, γ_ϕ, nicely captures the observed smaller steepness of the transition. Here, we have also assumed a small thermal photon number of the environment, $\bar{n}_T = 0.1$. However, a closer inspection of the second-order moment, $|\langle a^2\rangle|$, indicates that γ_ϕ also leads to a significantly smaller saturation value of this quantity. To achieve a better fitting between the simulation and the experiments, one may consider to include the second-order processes into the simulation, which has been neglected for deriving Supplementary Eq. (2). Here, we consider the two-photon drive, $\xi_2\left(a^2 + a^{\dagger 2}\right)$, and correspondingly the two-photon loss, $(\gamma_2/2)\,D\left[a^2\right]$. These higher-order processes should be weak, such that the parameters, ξ_2 and γ_2, are assumed to be smaller than ξ and γ, respectively. We achieve a quantitive agreement between theory and experiment for $\xi_2 = 0.3\xi$ and $\gamma_2 = 0.1\gamma$. These results demonstrate that we are able to achieve a consistent interpretation of our experimental results by introducing dephasing and two-photon process. Nevertheless, we emphasize that the conclusions drawn from our experiment are either insensitive to the dephasing rate, such as the hysteretic behavior (Fig. 1 of the main text), two-stage relaxation process (Fig. 2 of the main text), or based on the high-frequency measurements where the dephasing rate is much smaller than the energy dissipation, such as the increasingly sharp transition step with scaling factor N (Fig. 3 of the main text) and the quantum state tomography results (Fig. 4 of the main text).

---

[1] M. Leib, F. Deppe, A. Marx, R. Gross, and M. J. Hartmann, Networks of nonlinear superconducting transmission line resonators, New J. Phys. **14**, 075024 (2012).
[2] M. Fischer, Q.-M. Chen, C. Besson, P. Eder, J. Goetz, S. Pogorzalek, M. Renger, E. Xie, M. J. Hartmann, K. G. Fedorov, A. Marx, F. Deppe, and R. Gross, In situ tunable nonlinearity and competing signal paths in coupled superconducting resonators, Phys. Rev. B **103**, 094515 (2021).
[3] Q.-M. Chen, M. Pfeiffer, M. Partanen, F. Fesquet, K. E. Honasoge, F. Kronowetter, Y. Nojiri, M. Renger, K. G. Fedorov, A. Marx, F. Deppe, and R. Gross, Scattering coefficients of superconducting microwave resonators. I. Transfer matrix approach, Phys. Rev. B **106**, 214505 (2022).
[4] Q.-M. Chen, M. Partanen, F. Fesquet, K. E. Honasoge, F. Kronowetter, Y. Nojiri, M. Renger, K. G. Fedorov, A. Marx, F. Deppe, and R. Gross, Scattering coefficients of superconducting microwave resonators. II. System-bath approach, Phys. Rev. B **106**, 214506 (2022).
[5] S. Haroche and J.-M. Raimond, *Exploring the Quantum: Atoms, Cavities, and Photons* (Oxford University Press, 2006).
[6] P. D. Drummond and D. F. Walls, Quantum theory of optical bistability. i. nonlinear polarisability model, J. Phys. A: Math. Gen. **13**, 725 (1980).
[7] K. Vogel and H. Risken, Quasiprobability distributions in dispersive optical bistability, Phys. Rev. A **39**, 4675 (1989).
[8] K. V. Kheruntsyan, Wigner function for a driven anharmonic oscillator, J. Opt. B: Quantum Semiclass. **1**, 225 (1999).
[9] W. Casteels, R. Fazio, and C. Ciuti, Critical dynamical properties of a first-order dissipative phase transition, Phys. Rev. A **95**, 012128 (2017).
[10] M. I. Dykman, Critical exponents in metastable decay via quantum activation, Phys. Rev. E **75**, 011101 (2007).
[11] I. Serban, M. I. Dykman, and F. K. Wilhelm, Relaxation of a qubit measured by a driven duffing oscillator, Phys. Rev. A **81**, 022305 (2010).
[12] L. Guo, Z. Zheng, X.-Q. Li, and Y. Yan, Dynamic quantum tunneling in mesoscopic driven duffing oscillators, Phys. Rev. E **84**, 011144 (2011).
[13] L. D. Landau and E. M. Lifshitz, *Mechanics*, 3rd ed., Course of Theoretical Physics Series, Vol. 1 (Butterworth-Heinemann, 1976).
[14] M. Watanabe, K. Inomata, T. Yamamoto, and J.-S. Tsai, Power-dependent internal loss in josephson bifurcation amplifiers, Phys. Rev. B **80**, 174502 (2009).
[15] A. Palacios-Laloy, F. Nguyen, F. Mallet, P. Bertet, D. Vion, and D. Esteve, Tunable resonators for quantum circuits, J. Low Temp. Phys. **151**, 1034 (2008).
[16] M. Sandberg, C. M. Wilson, F. Persson, T. Bauch, G. Johansson, V. Shumeiko, T. Duty, and P. Delsing, Tuning the field in a microwave resonator faster than the photon lifetime, Appl. Phys. Lett. **92**, 203501 (2008).
[17] C. M. Caves, Quantum limits on noise in linear amplifiers, Phys. Rev. D **26**, 1817 (1982).
[18] M. Mariantoni, E. P. Menzel, F. Deppe, M. A. Araque Caballero, A. Baust, T. Niemczyk, E. Hoffmann, E. Solano, A. Marx, and R. Gross, Planck spectroscopy and quantum noise of microwave beam splitters, Phys. Rev. Lett. **105**, 133601 (2010).
[19] M. Renger, S. Pogorzalek, Q. Chen, Y. Nojiri, K. Inomata, Y. Nakamura, M. Partanen, A. Marx, R. Gross, F. Deppe, and

K. G. Fedorov, Beyond the standard quantum limit for parametric amplification of broadband signals, npj Quantum Inf. **7**, 1 (2021).

[20] E. P. Menzel, F. Deppe, M. Mariantoni, M. A. Araque Caballero, A. Baust, T. Niemczyk, E. Hoffmann, A. Marx, E. Solano, and R. Gross, Dual-path state reconstruction scheme for propagating quantum microwaves and detector noise tomography, Phys. Rev. Lett. **105**, 100401 (2010).

[21] E. P. Menzel, R. Di Candia, F. Deppe, P. Eder, L. Zhong, M. Ihmig, M. Haeberlein, A. Baust, E. Hoffmann, D. Ballester, K. Inomata, T. Yamamoto, Y. Nakamura, E. Solano, A. Marx, and R. Gross, Path entanglement of continuous-variable quantum microwaves, Phys. Rev. Lett. **109**, 250502 (2012).

[22] L. Zhong, E. P. Menzel, R. D. Candia, P. Eder, M. Ihmig, A. Baust, M. Haeberlein, E. Hoffmann, K. Inomata, T. Yamamoto, Y. Nakamura, E. Solano, F. Deppe, A. Marx, and R. Gross, Squeezing with a flux-driven josephson parametric amplifier, New J. Phys. **15**, 125013 (2013).

[23] K. G. Fedorov, L. Zhong, S. Pogorzalek, P. Eder, M. Fischer, J. Goetz, E. Xie, F. Wulschner, K. Inomata, T. Yamamoto, Y. Nakamura, R. Di Candia, U. Las Heras, M. Sanz, E. Solano, E. P. Menzel, F. Deppe, A. Marx, and R. Gross, Displacement of propagating squeezed microwave states, Phys. Rev. Lett. **117**, 020502 (2016).

[24] J. Goetz, S. Pogorzalek, F. Deppe, K. G. Fedorov, P. Eder, M. Fischer, F. Wulschner, E. Xie, A. Marx, and R. Gross, Photon statistics of propagating thermal microwaves, Phys. Rev. Lett. **118**, 103602 (2017).

[25] K. G. Fedorov, S. Pogorzalek, U. L. Heras, M. Sanz, P. Yard, P. Eder, M. Fischer, J. Goetz, E. Xie, K. Inomata, Y. Nakamura, R. D. Candia, E. Solano, A. Marx, F. Deppe, and R. Gross, Finite-time quantum entanglement in propagating squeezed microwaves, Sci. Rep. **8**, 6416 (2018).

[26] S. Pogorzalek, K. G. Fedorov, M. Xu, A. Parra-Rodriguez, M. Sanz, M. Fischer, E. Xie, K. Inomata, Y. Nakamura, E. Solano, A. Marx, F. Deppe, and R. Gross, Secure quantum remote state preparation of squeezed microwave states, Nat. Comm. **10**, 1604 (2019).

[27] K. G. Fedorov, M. Renger, S. Pogorzalek, R. D. Candia, Q.-M. Chen, Y. Nojiri, K. Inomata, Y. Nakamura, M. Partanen, A. Marx, R. Gross, and F. Deppe, Experimental quantum teleportation of propagating microwaves, Sci. Adv. **7**, eabk0891 (2021).

[28] F. R. Ong, M. Boissonneault, F. Mallet, A. Palacios-Laloy, A. Dewes, A. C. Doherty, A. Blais, P. Bertet, D. Vion, and D. Esteve, Circuit qed with a nonlinear resonator: ac-stark shift and dephasing, Phys. Rev. Lett. **106**, 167002 (2011).

[29] M. S. Kim and N. Imoto, Phase-sensitive reservoir modeled by beam splitters, Phys. Rev. A **52**, 2401 (1995).

[30] M. S. Kim, Quasiprobability functions measured by photon statistics of amplified signal fields, Phys. Rev. A **56**, 3175 (1997).

[31] C. Eichler, D. Bozyigit, and A. Wallraff, Characterizing quantum microwave radiation and its entanglement with superconducting qubits using linear detectors, Phys. Rev. A **86**, 032106 (2012).

[32] M. Hofheinz, E. Weig, M. Ansmann, R. C. Bialczak, E. Lucero, M. Neeley, O. AD, H. Wang, J. M. Martinis, and A. Cleland, Generation of fock states in a superconducting quantum circuit, Nature **454**, 310 (2008).

[33] M. Hofheinz, H. Wang, M. Ansmann, R. C. Bialczak, E. Lucero, M. Neeley, O. AD, D. Sank, J. Wenner, J. M. Martinis, and A. Cleland, Synthesizing arbitrary quantum states in a superconducting resonator, Nature **459**, 546 (2009).

[34] Y. Shalibo, R. Resh, O. Fogel, D. Shwa, R. Bialczak, J. M. Martinis, and N. Katz, Direct wigner tomography of a superconducting anharmonic oscillator, Phys. Rev. Lett. **110**, 100404 (2013).

[35] G. Kirchmair, B. Vlastakis, Z. Leghtas, S. E. Nigg, H. Paik, E. Ginossar, M. Mirrahimi, L. Frunzio, S. M. Girvin, and R. J. Schoelkopf, Observation of quantum state collapse and revival due to the single-photon kerr effect, Nature **495**, 205 (2013).

[36] J. Bajer, A. Miranowicz, and M. Andrzejewski, Quantum noise and mixedness of a pumped dissipative non-linear oscillator, J. Opt. B: Quantum Semiclass. **6**, 387 (2004).

[37] B. Dubost, M. Koschorreck, M. Napolitano, N. Behbood, R. J. Sewell, and M. W. Mitchell, Efficient quantification of non-gaussian spin distributions, Phys. Rev. Lett. **108**, 183602 (2012).
