# Supplementary figures and images for: Quantum behavior of the Duffing oscillator at the dissipative phase transition
Source: Nat Commun. 2023 May 20;14:2896. doi: 10.1038/s41467-023-38217-x (PMC10199948; doi:10.1038/s41467-023-38217-x)

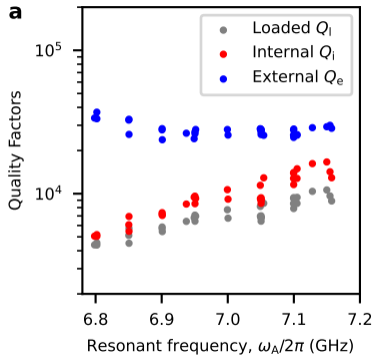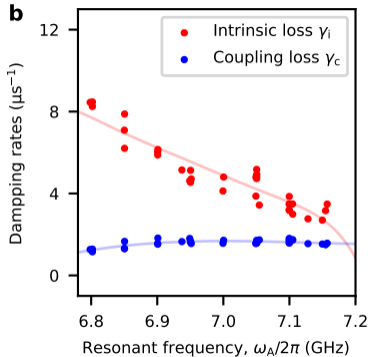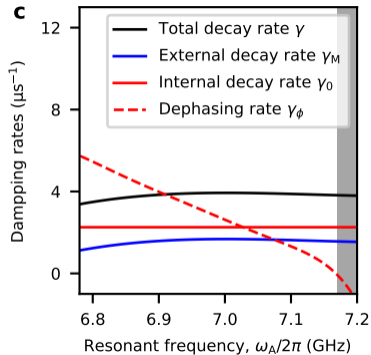

Supplement: Supplementary file 3 — Source Data file [file 41467_2023_38217_MOESM3_ESM.zip › Source_Data/Supplementary_Fig4/Fig_open.pdf]

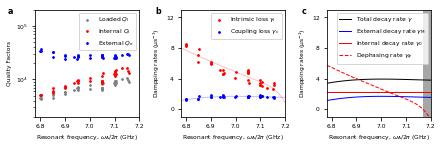

Supplement: Supplementary file 3 — Source Data file [file 41467_2023_38217_MOESM3_ESM.zip › Source_Data/Supplementary_Fig4/Fig_open.jpg]

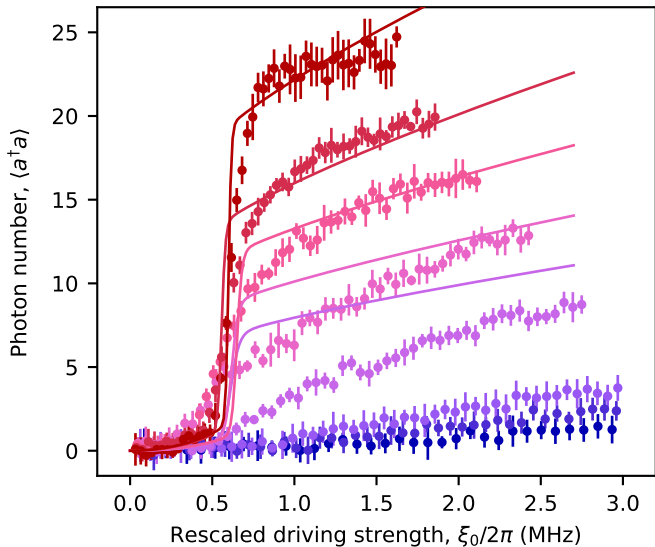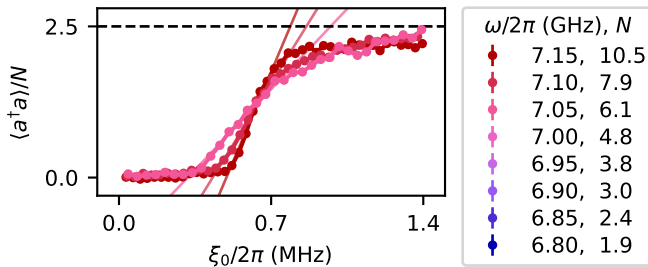

Supplement: Supplementary file 3 — Source Data file [file 41467_2023_38217_MOESM3_ESM.zip › Source_Data/Main_Fig3/Fig_transition.pdf]

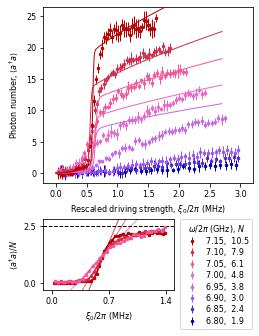

Supplement: Supplementary file 3 — Source Data file [file 41467_2023_38217_MOESM3_ESM.zip › Source_Data/Main_Fig3/Fig_transition.jpg]

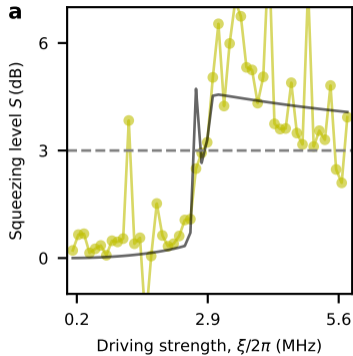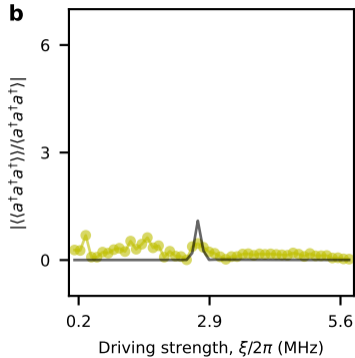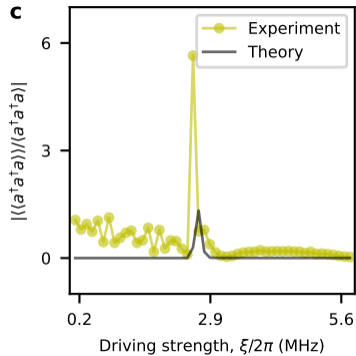

Supplement: Supplementary file 3 — Source Data file [file 41467_2023_38217_MOESM3_ESM.zip › Source_Data/Supplementary_Fig11/Res20210119_1/Fig_squeezing.pdf]

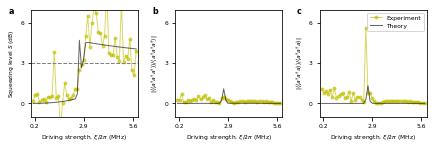

Supplement: Supplementary file 3 — Source Data file [file 41467_2023_38217_MOESM3_ESM.zip › Source_Data/Supplementary_Fig11/Res20210119_1/Fig_squeezing.jpg]

$G=108.6 \text{ dB}$ ,  $n=7.26 \text{ (s} \cdot \text{Hz)}^{-1}$

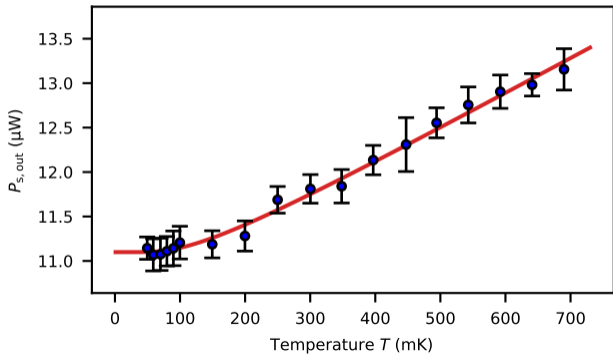

Supplement: Supplementary file 3 — Source Data file [file 41467_2023_38217_MOESM3_ESM.zip › Source_Data/Supplementary_Fig5/Res20210106_1/Fig_output.pdf]

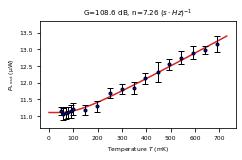

Supplement: Supplementary file 3 — Source Data file [file 41467_2023_38217_MOESM3_ESM.zip › Source_Data/Supplementary_Fig5/Res20210106_1/Fig_output.jpg]

0.0  $|\langle a \rangle|$  3.8 0.0  $\langle a^\dagger a \rangle$  13.9

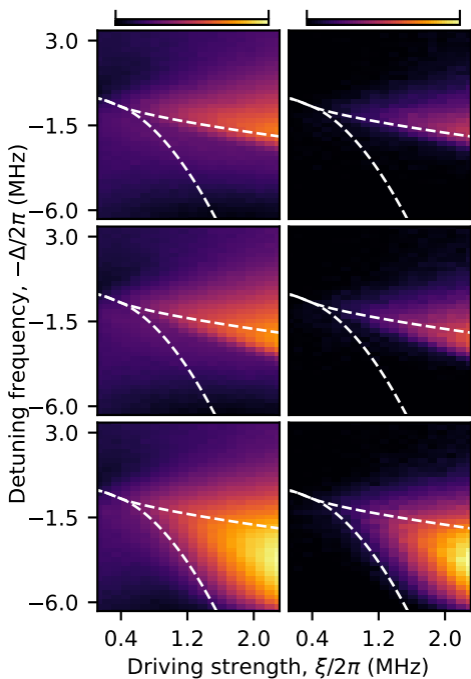

Supplement: Supplementary file 3 — Source Data file [file 41467_2023_38217_MOESM3_ESM.zip › Source_Data/Main_Fig1b/Res20201226_1/Fig_hysteresis_b.pdf]

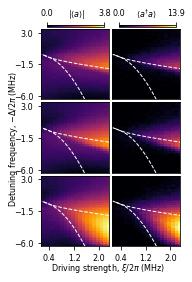

Supplement: Supplementary file 3 — Source Data file [file 41467_2023_38217_MOESM3_ESM.zip › Source_Data/Main_Fig1b/Res20201226_1/Fig_hysteresis_b.jpg]

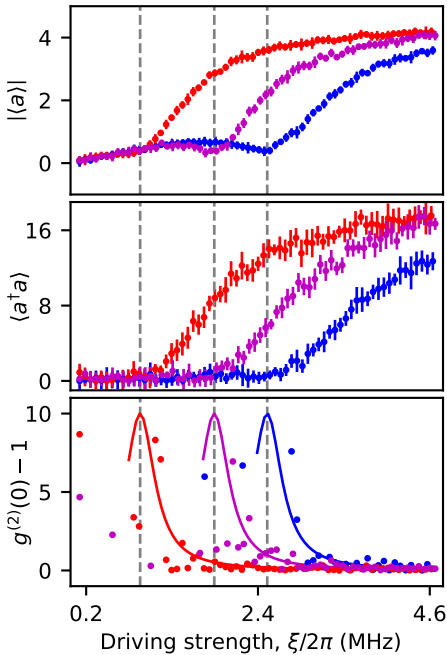

Supplement: Supplementary file 3 — Source Data file [file 41467_2023_38217_MOESM3_ESM.zip › Source_Data/Main_Fig1c/Res20210202_13/Fig_hysteresis_c.pdf]

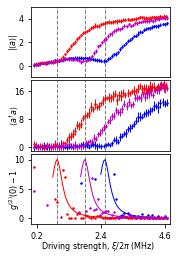

Supplement: Supplementary file 3 — Source Data file [file 41467_2023_38217_MOESM3_ESM.zip › Source_Data/Main_Fig1c/Res20210202_13/Fig_hysteresis_c.jpg]

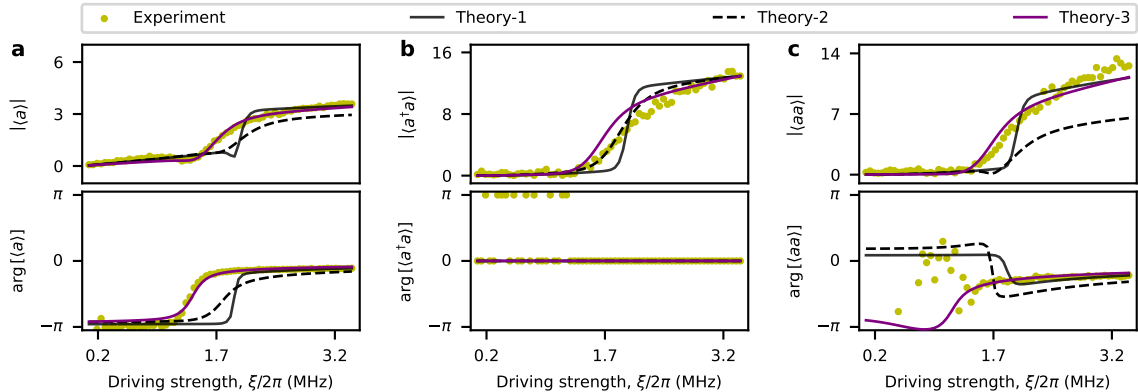

Supplement: Supplementary file 3 — Source Data file [file 41467_2023_38217_MOESM3_ESM.zip › Source_Data/Supplementary_Fig12/Res20210202_13/Fig_dephasing.pdf]

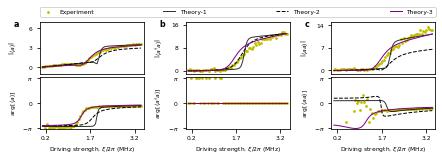

Supplement: Supplementary file 3 — Source Data file [file 41467_2023_38217_MOESM3_ESM.zip › Source_Data/Supplementary_Fig12/Res20210202_13/Fig_dephasing.jpg]

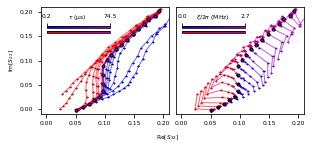

Supplement: Supplementary file 3 — Source Data file [file 41467_2023_38217_MOESM3_ESM.zip › Source_Data/Supplementary_Fig8/Res20201229_1/Fig_loop.jpg]

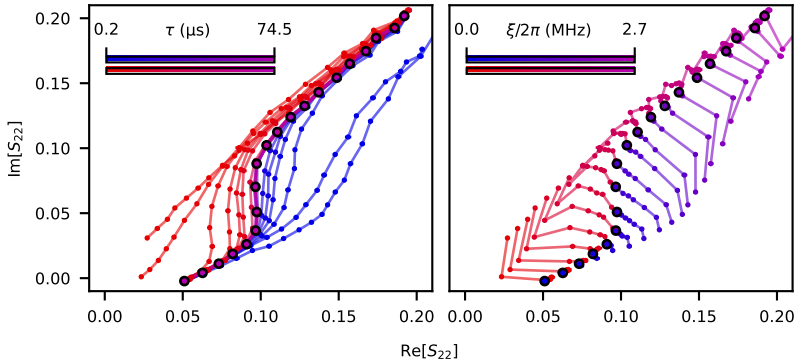

Supplement: Supplementary file 3 — Source Data file [file 41467_2023_38217_MOESM3_ESM.zip › Source_Data/Supplementary_Fig8/Res20201229_1/Fig_loop.pdf]

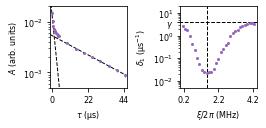

Supplement: Supplementary file 3 — Source Data file [file 41467_2023_38217_MOESM3_ESM.zip › Source_Data/Main_Fig2_AND_Supplementary_Fig9/Res20200901_1/Fig_metastability_b_c.jpg]

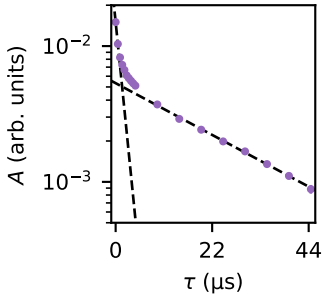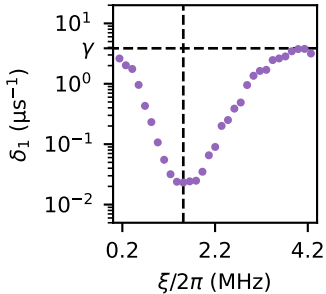

Supplement: Supplementary file 3 — Source Data file [file 41467_2023_38217_MOESM3_ESM.zip › Source_Data/Main_Fig2_AND_Supplementary_Fig9/Res20200901_1/Fig_metastability_b_c.pdf]

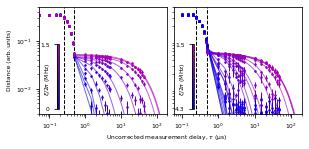

Supplement: Supplementary file 3 — Source Data file [file 41467_2023_38217_MOESM3_ESM.zip › Source_Data/Main_Fig2_AND_Supplementary_Fig9/Res20200901_1/Fig_gap.jpg]

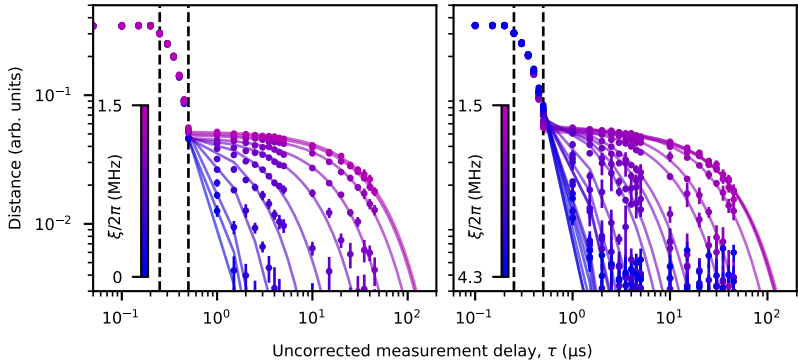

Supplement: Supplementary file 3 — Source Data file [file 41467_2023_38217_MOESM3_ESM.zip › Source_Data/Main_Fig2_AND_Supplementary_Fig9/Res20200901_1/Fig_gap.pdf]

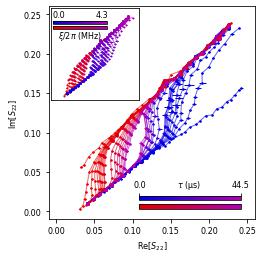

Supplement: Supplementary file 3 — Source Data file [file 41467_2023_38217_MOESM3_ESM.zip › Source_Data/Main_Fig2_AND_Supplementary_Fig9/Res20200901_1/Fig_metastability_a.jpg]

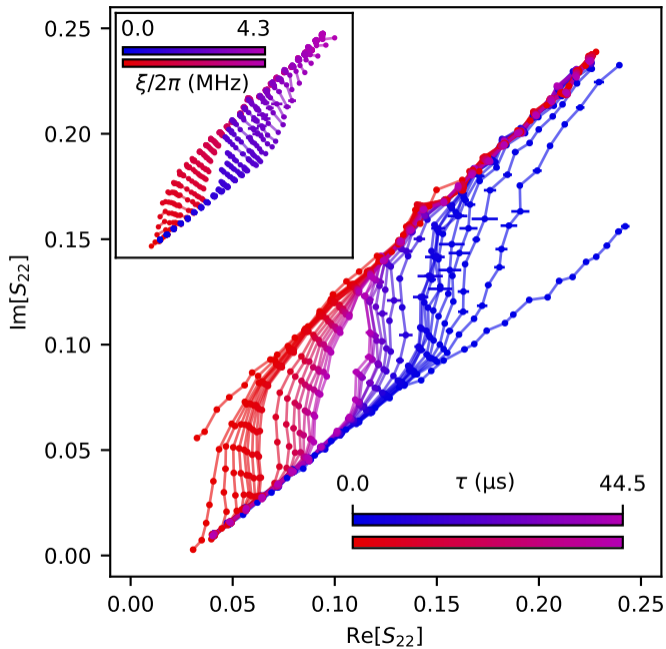

Supplement: Supplementary file 3 — Source Data file [file 41467_2023_38217_MOESM3_ESM.zip › Source_Data/Main_Fig2_AND_Supplementary_Fig9/Res20200901_1/Fig_metastability_a.pdf]

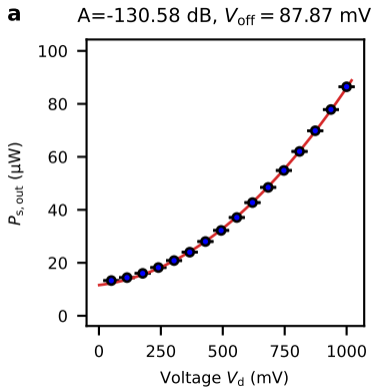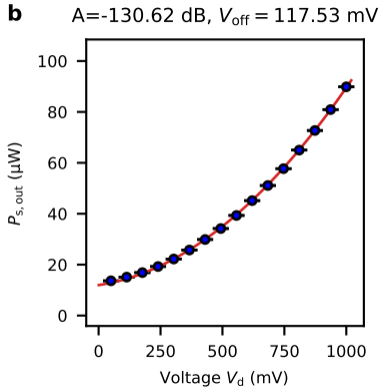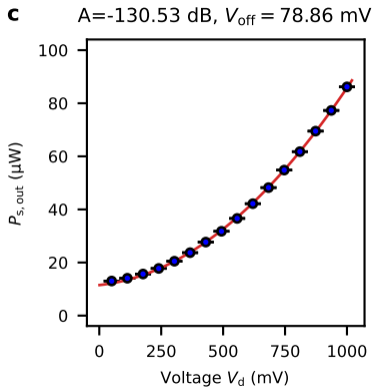

Supplement: Supplementary file 3 — Source Data file [file 41467_2023_38217_MOESM3_ESM.zip › Source_Data/Supplementary_Fig6/Res20210106_3/Fig_input.pdf]

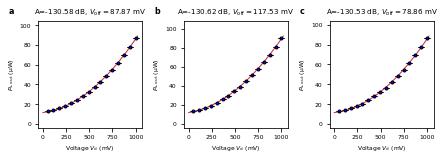

Supplement: Supplementary file 3 — Source Data file [file 41467_2023_38217_MOESM3_ESM.zip › Source_Data/Supplementary_Fig6/Res20210106_3/Fig_input.jpg]

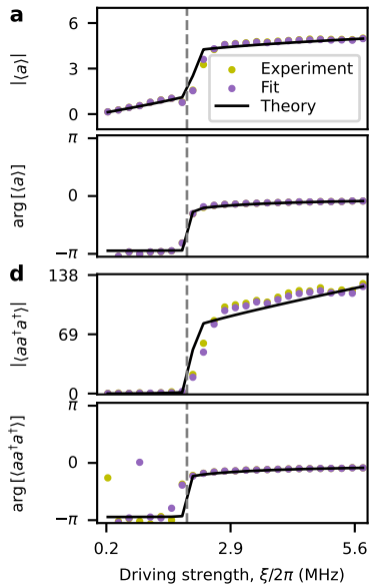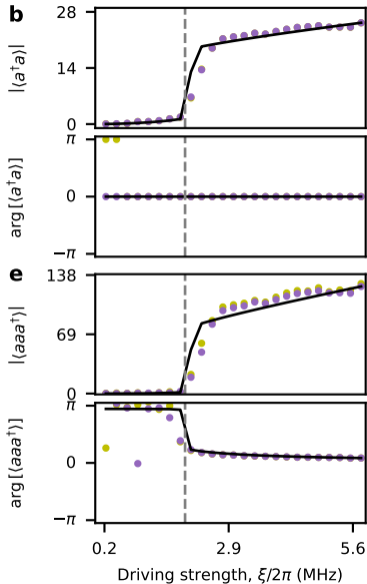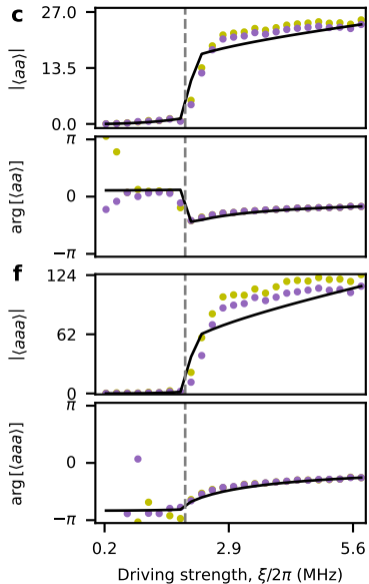

Supplement: Supplementary file 3 — Source Data file [file 41467_2023_38217_MOESM3_ESM.zip › Source_Data/Main_Fig4_AND_Supplementary_Fig10/Res20210128_3/Fig_moments.pdf]

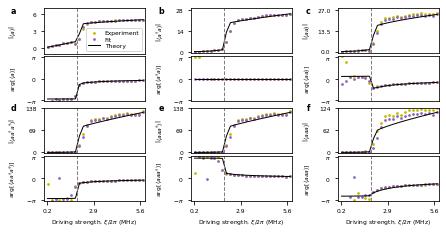

Supplement: Supplementary file 3 — Source Data file [file 41467_2023_38217_MOESM3_ESM.zip › Source_Data/Main_Fig4_AND_Supplementary_Fig10/Res20210128_3/Fig_moments.jpg]

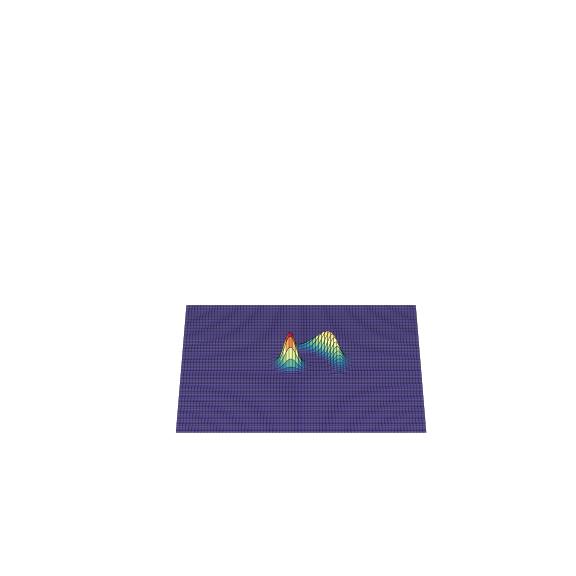

Supplement: Supplementary file 3 — Source Data file [file 41467_2023_38217_MOESM3_ESM.zip › Source_Data/Main_Fig4_AND_Supplementary_Fig10/Res20210128_3/Fig_fancy.jpg]

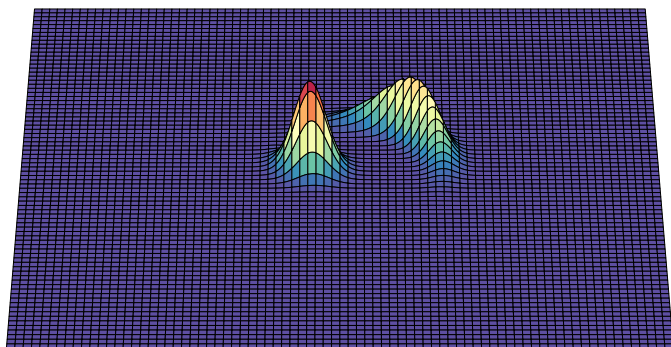

Supplement: Supplementary file 3 — Source Data file [file 41467_2023_38217_MOESM3_ESM.zip › Source_Data/Main_Fig4_AND_Supplementary_Fig10/Res20210128_3/Fig_fancy.pdf]

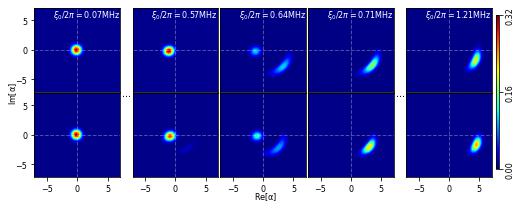

Supplement: Supplementary file 3 — Source Data file [file 41467_2023_38217_MOESM3_ESM.zip › Source_Data/Main_Fig4_AND_Supplementary_Fig10/Res20210128_3/Fig_tomography.jpg]

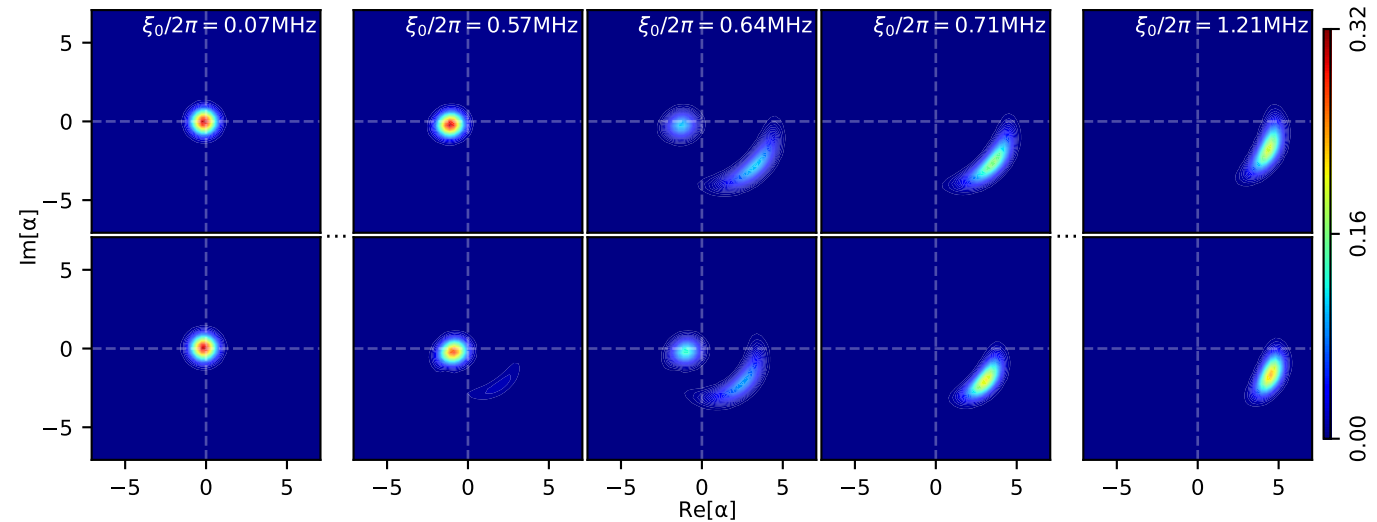

Supplement: Supplementary file 3 — Source Data file [file 41467_2023_38217_MOESM3_ESM.zip › Source_Data/Main_Fig4_AND_Supplementary_Fig10/Res20210128_3/Fig_tomography.pdf]

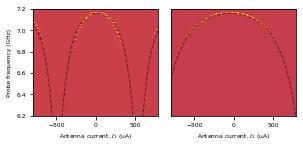

Supplement: Supplementary file 3 — Source Data file [file 41467_2023_38217_MOESM3_ESM.zip › Source_Data/Supplementary_Tab1/Res20210101_1/Fig_closed.jpg]

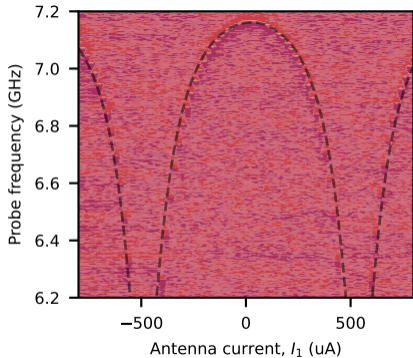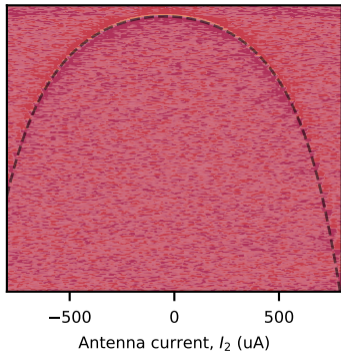

Supplement: Supplementary file 3 — Source Data file [file 41467_2023_38217_MOESM3_ESM.zip › Source_Data/Supplementary_Tab1/Res20210101_1/Fig_closed.pdf]
